# The application of cerebellar repetitive transcranial magnetic stimulation on aphasia after stroke: A randomized controlled trial protocol

**DOI:** 10.1371/journal.pone.0354837

**Published:** 2026-08-10

**Authors:** Qi Liu, Yang Liu, Yumei Zhang

**Affiliations:** 1 Department of Neurology, Beijing Tiantan Hospital, Capital Medical University, Beijing, China; 2 Department of Rehabilitation, Beijing Tiantan Hospital, Capital Medical University, Beijing, China; 3 China National Clinical Research Center for Neurological Diseases, Beijing Tiantan Hospital, Capital Medical University, Beijing, China; PLoS ONE, UNITED STATES OF AMERICA

## Abstract

**Objectives:**

Non-invasive brain stimulation has emerged as a safe, pain-free, and cost-effective method for post-stroke aphasia (PSA) rehabilitation. However, the ideal site for stimulation is yet to be determined. The cerebellum has demonstrated its role in language function though the application of repetitive transcranial magnetic stimulation (rTMS) targeting the cerebellum for PSA has not been well-documented. This study aims to assess the short- and long-term efficacy of rTMS on the right cerebellum in enhancing language recovery in patients with PSA in the subacute phase, and to explore its neuropsychological and neural network mechanisms as well.

**Methods:**

Our study will recruit sixty-six PSA patients, who will be randomly divided into either a real or a sham rTMS group with equal distribution. The real rTMS group (n  =  33) will receive rTMS treatment over the right cerebellar hemisphere along with speech-language therapy (SLT), while the sham group (n  =  33) will receive sham stimulation and SLT. We will assess the outcomes at five intervals: before rTMS treatment, 2, 12, and 24 weeks, and 1-year post-treatment. The primary outcome in this research is the change in performance of the West Aphasia Battery from baseline to the final follow-up. Secondary outcomes include alterations in the structure, function, and perfusion neuroimaging data, as well as changes in non-verbal cognitive functions, measured using the Loewenstein Occupational Therapy Cognitive Assessment over time and between the groups. Any adverse event related to rTMS therapy will also be recorded. The results of this trial will provide insights into whether cerebellar rTMS stimulation (1) improves language function recovery in subacute phase PSA patients; (2) strengthens the functional connection of the cerebro-cerebellar network, and (3) enhances aphasia treatment outcomes by improving non-linguistic cognitive functions. We commenced patient recruitment for this trial in June 2026, and it is currently in progress.

**Registration** This project was prospectively registered at the Chinese Clinical Trial Registry (ChiCTR2600125453).

## Introduction

Post-stroke aphasia (PSA), often results from brain lesions impacting cortical and subcortical structures, is characterized by difficulties in speech, comprehension, repetition, and naming, leading to extended hospital stays, diminished quality of life, and increased mortality [1]. Currently, the go-to treatment protocol for PSA is speech-language therapy (SLT), which has demonstrated beneficial effects on language function [2–4]. However, not all PSA patients have access to this therapy, and not all benefit from it. Despite SLT, individuals with aphasia frequently experience persistent communication challenges [3,5]. Thus, the development of additional, more effective therapeutic approaches for aphasia treatment is crucial. Researchers are exploring non-invasive brain stimulation (NIBS), a safe, painless, and cost-effective technique, as a potential enhancer for aphasia treatment [6–8]. The combination of NIBS with traditional therapy shows promise in aphasia recovery, as underscored by international guidelines [6,8].

Aphasia rehabilitation often targets the cerebrum, with most research focusing on stimulation of the left hemisphere due to the reactivation and utilization of the residual left-hemisphere tissue in language recovery [6,8–10]. Some studies apply inhibitory stimulation to the right hemisphere, based on the theory of inter-hemispheric disinhibition [11–13]. However, there are limitations to both approaches over the cerebral hemispheres [14]. Stimulating the left cerebral hemisphere can be problematic, as stroke often leaves areas with low electrical resistance due to filled with cerebrospinal fluid. This reduces the stimulation exposure on the targeted tissue [15–16]. Conversely, inhibiting the right hemisphere might also have negative effects, as evidence suggests that it may play a compensatory role in recovering language functions after a stroke [17–18]. Given the variability in lesion location and size, as well as the unpredictable brain reorganization, determining the optimal stimulation strategy at the cortical level is challenging [19]. Hence, it’s crucial to develop novel stimulation protocols targeting other significant areas involved in language processing, thereby improving the effectiveness of PSA treatment.

Recently, the cerebellum has gained attention as a new possible target for aphasia treatment [16,20–22]. Extensive research shows that the right cerebellum significantly contributes to a variety of language functions, such as word retrieval, verbal memory, language learning and semantic processing [23–24]. Apart from that, the cerebellum is generally far from the usual stroke locations linked to aphasia, reducing the chance of electrical current flow being affected by encephalomalacia [16]. Thus, this method works well for patients with cerebrum hemisphere strokes or aphasia related to bilateral brain strokes. Recent clinical studies have indicated that stimulating the right cerebellum with NIBS can enhance speech and language therapy in PSA patients [16,20–22], suggesting the cerebellum as an excellent site for neuromodulation in the management of PSA.

The cerebellum, with its unique plasticity mechanism and extensive connections to cortical areas, holds promise as a potential target for aphasia rehabilitation [25–26]. Cerebellar NIBS can induce cerebellar excitability changes and brain function modulation via the cerebellar-thalamic-cortical loop, as well as supporting the recovery of lost functions through inherent learning processes [27–30]. By enhancing functional connectivity between the right cerebellum and the cerebral cortical areas engaged in language processing, stimulation of the right cerebellum has demonstrated beneficial effects in enhancing SLT for language recovery [31]. Beyond its role in linguistic function, cerebellar NIBS also significantly contributes to other cognitive functions, notably in executive function that is tightly interconnected with language [31]. Our earlier research has underscored the significant correlation between non-linguistic cognitive impairments and language impairments in patients with PSA [32]. Thus, it is plausible to deduce that cerebellar stimulation could provide therapeutic benefits in aphasia by enhancing their non-linguistic cognitive functions, an aspect which remains unexplored in prior studies.

The majority of prior research, however, has focused on chronic stroke phase aphasia patients whose functional brain structures have already undergone irreversible alterations [4,33,34]. This could potentially explain the observed lack of significant effects of cerebellar NIBS on language recovery [22,35]. Recent guidelines indicate the safe and tolerable application of NIBS even in the sub-acute phase, with no severe adverse effects reported [36–39]. The current study protocol proposes to stimulate the right cerebellum with repetitive transcranial magnetic stimulation (rTMS) in order to enhance SLT for PSA patients in the sub-acute phase. The study’s primary objective is to assess the short- and long-term efficacy of cerebellar rTMS for aphasia recovery in these patients under a randomized, sham-controlled design. The secondary purpose is to examine alterations in functional connectivity between the cerebellum and language-related cortical regions pre- and post-treatment and elucidate the neural restructuring mechanism. Moreover, from a neuropsychological perspective, the study will investigate whether cerebellar stimulation can improve language symptoms through enhancing non-linguistic cognitive functions.

## Methods

### Study design

**Fig 1:**
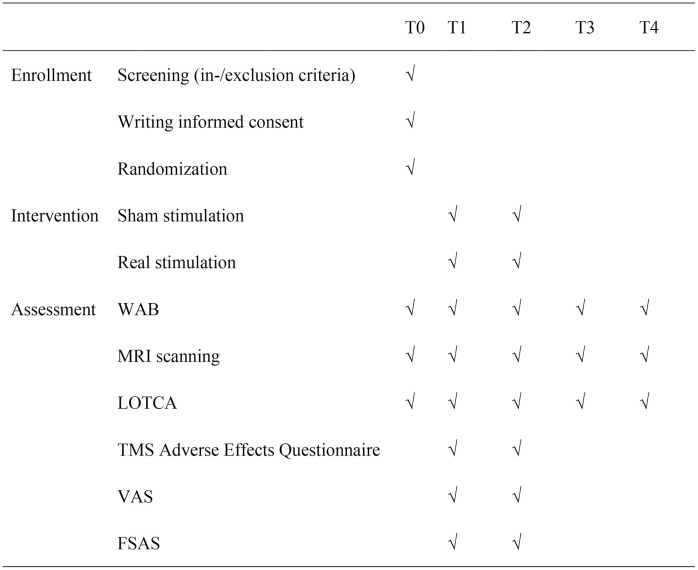
SPIRIT schedule. Abbreviation: WAB: West Aphasia Battery; MRI: Magnetic Resonance Imaging; LOTCA: The Loewenstein Occupational Therapy Cognitive Assessment; TMS: Transcranial Magnetic Stimulation; VAS: Visual Analog Scale; FSAS: Fatigue Self-Assessment Scale.

**Fig 2:**
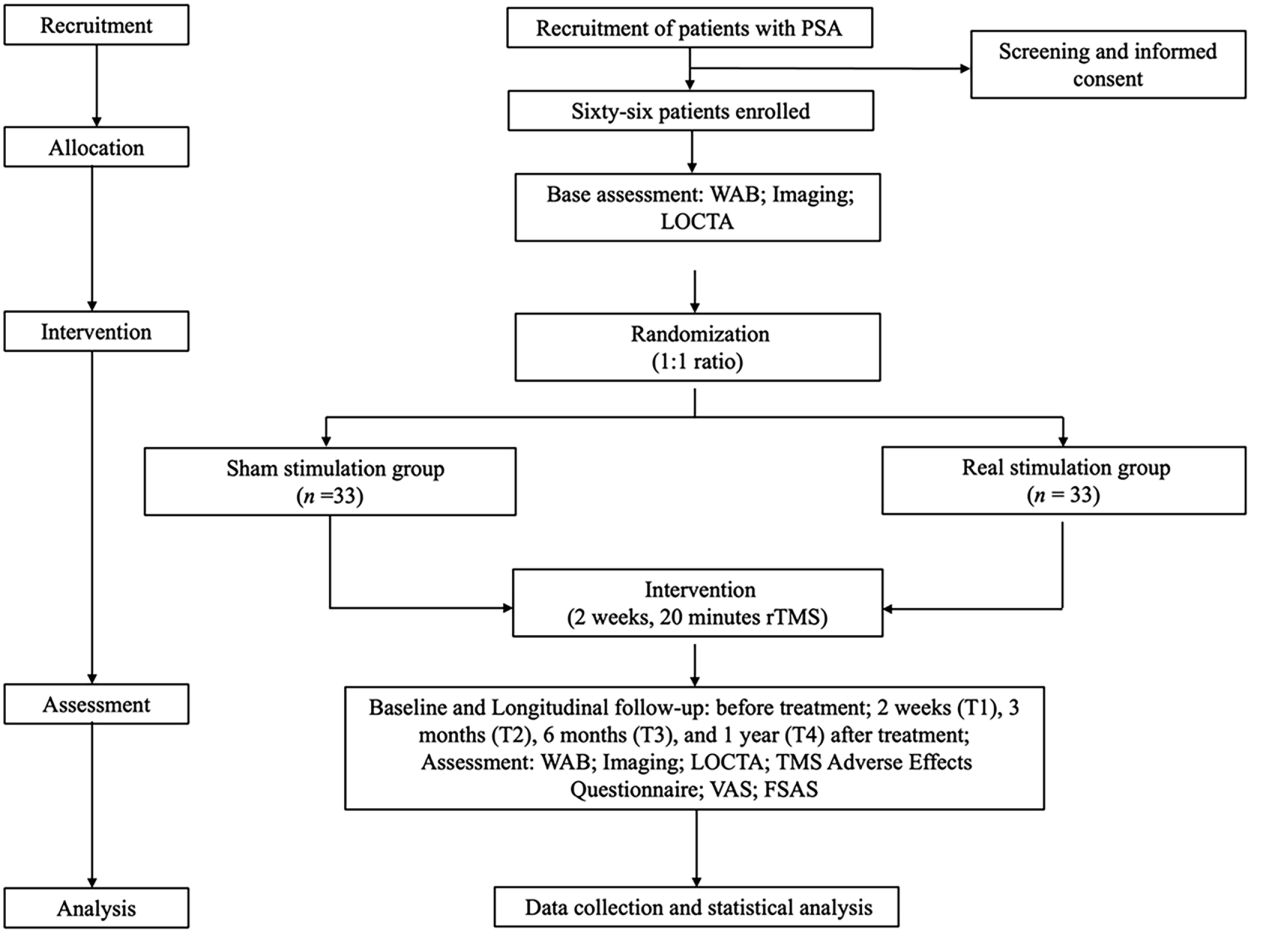
The flow chart of the study. Abbreviation: PSA: post-stroke aphasia; WAB: West Aphasia Battery; LOTCA: The Loewenstein Occupational Therapy Cognitive Assessment; rTMS: repetitive transcranial magnetic stimulation; VAS: Visual Analog Scale; FSAS: Fatigue Self-Assessment Scale.

This is a randomized, double-blinded, sham-controlled and single-center clinical study (Registry: Chinese Clinical Trial Registry; Registration number: ChiCTR2600125453). Patient with PSA will be recruited and will be randomized 1:1 into two groups: the sham and real stimulation group. Patient in the real stimulation group will receive rTMS targeting the right cerebellum, as well as conventional language rehabilitation training. While the sham stimulation will be implemented in the sham group. Language and cognitive function evaluation and neuroimaging data will be collected before rTMS treatment (T0) and at 2 weeks (T1), 3 months (T2), 6 months (T3), and 1 year (T4) after treatment (Please see more details in Fig 1 and Fig 2). The trial protocol conforms to the Standard Protocol Items: Recommendations for Interventional Trials (SPIRIT) Checklist (S1 Checklist) [40]. All participants have provided signed informed consent (S2 File). The study will adhere to the guidelines set forth in the Declaration of Helsinki.

### Recruitment

Participants were recruited in the department of neurology and rehabilitation in Beijing Tiantan Hospital. Patients will be recruited for the trial through leaflet distribution and posting recruitment information on the bulletin board. Participation is voluntary, and individuals will have a complete understanding of the trial’s details before enrolling. The attending physician will collect data on medical history, neuroimaging, and scale assessment results.

### Eligibility criteria

#### Inclusion criteria.

To be eligible for the study, participants must be 18 years or older, right-handed, modern standard Chinese Mandarin-speaking (primary language), with more than 6 years of educational level. The study will collect first-onset stroke aphasia patients with the National Institute of Health stroke scale (NIHSS) score ≤ 3 and the lesion located in the left cerebral hemisphere. Patients will be diagnosed according to current clinical diagnostic criteria of ischemic stroke based on informant report, neurological examination, and brain neuroimaging [41]. And aphasia will be diagnosed by the western aphasia battery (WAB) with the standard of aphasia quotient (AQ)< 93.8 [42]. (See the detailed description about the scale in the primary outcome part)

#### Exclusion criteria.

Exclusion criteria comprise pre-existing language barrier or aphasia caused by other disorders. History of other neurological diseases (e.g., cerebral small vessel disease, Alzheimer’s disease), mental illnesses (e.g., schizophrenia), other system diseases (e.g., hypothyroidism) which may cause cognitive impairments and consuming diseases or general poor health are further exclusion criteria for all participants. Furthermore, participants who are unable to cooperate in completing the assessment scales and have contraindications for rTMS therapy and MRI examination will not be recruited.

### Sample size

The sample size was estimated using G*power of 3.1.9.6 (www.gpower.hhu.de) [43–44], with the following parameters: effect size (*d*) = 0.86, α = 0.05 (two tails), power (1–β) = 90%, and allocation ratio n2/n1 = 1. The effect size was determined based on the result of our pilot study. The sample size in the current study should be 60 (30 in each group) after calculation. Considering a 10% dropout rate, a total of 66 patients are aimed to be recruited finally.

### Randomization and blinding

After obtaining informed consent, a random sequence will be generated by using the computer. A total of 66 participants will then be assigned randomly to the two groups (real or sham rTMS group) at a 1:1 ratio. Blinded grouping will be implemented using sealed envelopes with numbers. Both participants and the physiotherapists responsible for administering language rehabilitation training and rTMS therapy will be blinded to the treatment allocation.

### Interventions

Participants will receive rTMS treatment by a same magnetic stimulator (model: YRD CCY-I, Wuhan Yiruide medical equipment New Technology Co., Ltd.). The targeted location is the right cerebellum, specifically 1 cm below the occipital tuberosity and 3 cm above the right paraspinal opening. rTMS treatment takes place at this spot utilizing an 8-shaped coil (9.2 cm in outer diameter), positioned fixedly and tangent to the scalp. The stimulation parameters include a frequency of 5 Hz, intensity set at 110% of the resting motor threshold (RMT) of the contralateral cerebral hemisphere, a duration of 5s, 25 pulses, a string interval of 20s, a session time of 20 min, 48 repetitions, totaling 1200 pulses [45–46]. In the sham stimulation group, the body surface positioning points and stimulation parameters are identical to those in the real rTMS group. However, the coil is positioned perpendicular to the scalp. Participants will receive treatments once a day for 20 minutes, five days a week, over a two-week period. Additionally, both groups will undergo conventional language rehabilitation training, with each session lasting 30 minutes and conducted twice a day.

### Outcome measurement

Outcome measures will be assessed across five time points: prior to rTMS treatment (T0), and at 2 weeks (T1), 3 months (T2), 6 months (T3), and 1 year (T4) post-treatment. The primary outcome is the evaluation of language function. Neuroimaging data, along with evaluations of non-linguistic cognitive functions, will serve as secondary outcome measures (Table 1).

**Table 1:** Outcome measures.

Outcome measures
**Primary outcome measures** ● WAB for determining the presence of aphasia and assessing language dysfunction recovery and deterioration.
**Secondary outcome measures** ● Imaging data for evaluating the structural, function and perfusion alterations in brain area. ● LOTCA for assessing nonverbal cognitive function.
**Safety outcome** ● Incidence of adverse events. ● VAS for assessing headache symptoms. ● FSAS for assessing fatigue symptoms.

Abbreviation: WAB: West Aphasia Battery; LOTCA: The Loewenstein Occupational Therapy Cognitive Assessment; VAS: Visual Analog Scale; FSAS: Fatigue Self-Assessment Scale.

#### Primary outcome measurement.

The West Aphasia Battery (WAB) will be utilized to assess and monitor the improvement and deterioration of aphasia [42]. The assessment involves six language functions, which include spontaneous speech, auditory verbal comprehension, repetition, naming and word finding, reading, and writing. In the evaluation of aphasia, the AQ score is calculated to determine the severity of the condition. A higher AQ score indicates a milder form of aphasia and better language capabilities. The formula for calculating the AQ score is as follows: AQ = 2 × (spontaneous speech + auditory comprehension/20 + repetition/10 + naming/10). The total score is 100 points; higher scores suggest milder aphasia and improved language skills. AQ < 93.8 signifies aphasia.

#### Secondary outcome measurements.

All participants will undergo head MRI scans using a 3.0T, 48-channel MRI scanner (GE Healthcare, USA). T1-weighted sequences, T2-weighted sequences, and T2-fluid attenuated inversion recovery (FLAIR) will be obtained to identify and extract lesions. Resting-state functional MRI (rs-fMRI) will be conducted to evaluate local and global functional connectivity networks associated with language function enhancement. Moreover, diffusion tensor imaging (DTI) and Arterial Spin Labeling (ASL) sequences will be utilized to investigate changes in brain fibers and cerebral blood flow related to cerebellar stimulation on PSA, respectively. During the scanning procedure, rubber earplugs will be used to reduce noise, and foam pads will be utilized to secure the head position and minimize motion artifacts. In the rs-fMRI scanning session, participants will be instructed to close their eyes and remain awake.

In addition, the Loewenstein Occupational Therapy Cognitive Assessment (LOTCA) will be used to measure nonverbal cognitive function, encompassing domains of orientation, visual perception, spatial perception, motor praxis, visuo-motor organization, thinking operations and attention [47–48]. The total score ranges from 26 to 115 (the attention score was computed separately, ranging from 1 to 4). A lower score signifies more severe cognitive deficits.

#### Safety.

The rTMS Adverse Effects Questionnaire will be used to evaluate whether there are any adverse reactions such as burning, headache, tinnitus, anxiety, depression, and seizures in the two groups of patients during and after rTMS treatment. Headache and/or fatigue symptoms will prompt evaluations using the Visual Analog Scale (VAS) and Fatigue Self-Assessment Scale (FSAS) [49–50]. The safety assessments will be conducted after each intervention for all patients.

### Statistical analysis

#### Behavior data analysis.

SPSS 26.0 statistical software will be used to analyze behavior data (the performance of scale assessment which mentioned above). We will use the Intention-to-treat analysis in this study. Shapiro-Wilk test will be used to verify whether the data are normally distributed. For normally distributed measures, t-test and repeated-measures analysis of variances will be used to compare the behavior functions between the same group before and after treatment and between the real and sham rTMS group in the same phase. For non-normally distributed measures, the Mann-Whitney U Test and Kruskal-Wallis H test will be used for comparison across the conditions. P-value < 0.05 is considered significant (two-tailed).

#### Imaging data analysis.

MRI preprocessing and statistical analyses of all images will be implemented on MATLAB2021b (https://ww2.mathworks.cn) using statistical parametric mapping software (SPM12, https://www.fil.ion.ucl.ac.uk/spm/software/spm12/).

The processed data from DTI, rs-fMRI, and ASL will be employed to evaluate cerebellar stimulation’s effect on aphasia recovery in terms of structure, function, and perfusion. For the DTI data, variations in the cerebral-cerebellar loop’s structure will be identified between different groups and pre-/post treatment using Tract-Based Spatial Statistics (TBSS) analysis. With rs-fMRI, the analysis of amplitude of low-frequency fluctuations (ALFF), regional homogeneity (ReHo), and functional connectivity density (FCD) will be completed to assess the local and global functional connectivity inside the language networks. Using ASL sequences, the cerebral perfusion for every participant will be calculated, targeting the perfusion alteration, particularly the lesion area. One-way ANOVA will be performed to compare the imaging parameters among the groups (pre-vs. post-treatment, real vs. sham treatment). A linear regression analysis will be applied to establish the relationship between behavioral performance and the parameters. Any differences among these conditions are regarded as significant when the P-value is < 0.05 at the voxel level, with an FDR cluster-corrected P-value < 0.05 (two-tailed).

### Data collection and management

#### Data collection.

Data collection will be directly performed using either paper forms or specific evaluation tools. The content of each case report form (CRF) will align with and originate from the original files. The investigator bears the responsibility of ensuring the authenticity, thoroughness, and accuracy of all data. All items in the CRF must be completed, with no blanks or omissions; a slash will signify items with no records. Any corrections will be underscored, with the updated data annotated alongside the reason for the change then signed and dated by the employee making the correction. Erasing or overwriting the original record is strictly prohibited.

#### Data management.

Data originality, including documents, test reports, study summaries, and clinical trial outcomes will be archived in an electronic capture system. The archive room will ensure an organized storage and quick retrieval of all original data, test reports, protocols, and summaries. Participant privacy will be shielded by excluding their names from the CRF. Identification codes will be used by investigators to acknowledge participant identifiers and duly record them.

#### Data monitoring.

Upon review, the inspectors will assess adherence to all applicable rules, good clinical Practice, and the study protocol. They will verify the accuracy, completeness, and consistency of source documents of all CRFs while ensuring the absence of both data omissions and errors. This process involves cross-referential checks between the contents of the CRFs and the original files, effectively ensuring data uniformity. This process is known as source data verification.

#### Protocol violation.

The study protocol’s specified requisites must be meticulously followed. Whether intentional or accidental, any departure from the study protocol or good clinical practice will be classified as a protocol violation. If the deviations occur, the investigator or supervisor will complete a protocol violation record. Information regarding the discovery, process, reasons and corresponding resolution of the event will be documented, signed by the investigator, and submitted to the ethics committee.

#### Data release.

Approval from the principal investigator must be attained before any study-related articles or reports are published.

### Trial status

Participant recruitment commenced on June 1, 2026, and is expected to be completed by December 31, 2028. Data collection is expected to be completed by June 1, 2029. The research results are expected to be released by December 31, 2029.

### Ethics statement

This study was conducted in accordance with the Declaration of Helsinki (2013 revision) and approved by the Research Ethics Committee of Beijing Tiantan Hospital, Capital Medical University (Approval Number: KY2026-109-02; Date of Approval: 2026-05-11). For adult participants with aphasia, written informed consent was obtained from the participants themselves where their cognitive and communicative abilities permitted independent decision-making; for those with impaired capacity to provide informed consent, written informed consent was obtained from their legally authorized representatives. All participants and/or their representatives were fully informed of the study’s interventional nature, research aims, data collection procedures, and their right to withdraw from the study at any time without any adverse consequences. Throughout the entire research and data analysis process, strict confidentiality and anonymity of all participant data were guaranteed: all personally identifying information was fully de-identified and pseudonymized, and the de-identified data were securely stored in password-protected electronic devices with restricted access and locked physical archives.

### Registration

This study was prospectively registered at the Chinese Clinical Trial Registry (ChiCTR2600125453) on May 27, 2026.

## Discussion

### Trial significance

The complexity of language deficits makes aphasia rehabilitation a challenging process, however, it is of paramount importance in aiding individuals to effectively communicate and regain daily living independence [1–4]. NIBS has emerged as a beneficial adjunct to traditional speech-language therapy, despite the uncertainty surrounding the ideal stimulation site [6–8]. Notably, conventional supratentorial targets for NIBS present certain limitations [14–16]. According to previous studies [16,20–22], we posit a significant impact of cerebellar NIBS on PSA rehabilitation, supported by our prior research highlighting the association between aphasia symptoms and cerebellum network function alterations, and the role of non-linguistic cognitive function in PSA patients’ language recovery [32,51,52]. Our randomized, sham-controlled clinical trial will investigate the neural and behavioral effects of rTMS over the right cerebellum in PSA patients during the sub-acute phase, offering supportive evidence for the right cerebellum as an optimal target for language rehabilitation and elucidating its underlying mechanisms.

### Trial innovation

The proposed study protocol, featuring a rigorous randomized, parallel, controlled trial design, adheres to the methodological prerequisites of full concealment, randomization, and sham control. This approach is advantageous as it reduces bias and provides robust evidence to evaluate the effectiveness of cerebellar rTMS, thus, drawing a reliable conclusion [53].

Further, the choice of the sub-acute stroke phase as the intervention period is strategic. This period is in a neuroplasticity window making the brain particularly receptive to modulating intervention early in the recovery course [54]. Existing imaging studies on PSA generally agree that a brain activation shift, induced by tasks, transpires within the initial post-stroke weeks, followed by a normalization of activity patterns over several months [55–56]. Hence, we hypothesize that delivering the treatment during the subacute stroke phase could yield more significant effects than in the chronic phase. The effectiveness of the intervention will be assessed at various time points: pre-intervention, the second week’s end, the third and sixth months, and one-year post-intervention. This will enable a comparison of efficacy across different periods and the identification of the earliest effective treatment time.

Unlike many preceding studies that predominantly applied transcranial direct current stimulation (tDCS), this research will employ rTMS treatment. Compared to tDCS, rTMS creates resilient, enduring activation alterations in noticeably shorter timeframes [57]. Moreover, we will utilize a figure-of-8 coil on the cerebellum, proven to be a flat coil model with improved trial tolerances that can effectively stimulate the cerebellar cortex’s superficial layers [58].

Another unique point is that the current study will investigate the profound neuropsychological and neural network mechanisms influencing the impact of cerebellar rTMS on aphasia – a subject area relatively unexplored in previous research [20,22,59]. A multi-modal neuroimaging technique will be used to gauge modifications in the supratentorial areas’ structure, function, and perfusion, particularly the lesion part after treatment. This will also give insights into the relationship between these changes and language, along with non-verbal cognitive functions recovery.

### Expected conclusions

(1) Following treatment, language and non-verbal cognitive functions in the rTMS group are expected to exhibit better performance compared to those in the sham group.
(2) Changes in spontaneous neural activity within the fronto-thalamic-cerebellar circuit and enhancement in perfusion within the lesion site are anticipated following the stimulation therapy.

### Limitations and prospects of the trial

First, this study is constrained by its small sample size and single-center design. Additionally, the year-long follow-up period may lead to unpredictable dropout rates. Furthermore, despite efforts to match age and education levels between the sham and real rTMS groups, individual differences may result in distinct baseline conditions that could influence outcomes. The diverse mechanism of aphasia types poses another challenge, as subgroup analyses based on these types are impeded by the limited sample size. Future research should involve a prospective, multicenter, randomized controlled trial with a larger cohort to validate the current findings. While serving as a preliminary investigation, this study offers valuable insights and potential non-invasive stimulation approaches for enhancing language function in patients with PSA.

## Supporting information

S1 Checklist SPIRIT checklist. (DOC)

S2 File Informed consent form (Chinese). (PDF)

S3 File Study protocol (Chinese). (DOC)

S4 File Study protocol (English). (DOCX)

## References

[1] Lazar RM, Boehme AK. Aphasia as a predictor of stroke outcome. Curr Neurol Neurosci Rep. 2017;17(11):83. doi: 10.1007/s11910-017-0797-z 28929424 PMC13077792

[2] Bhogal SK, Teasell R, Speechley M. Intensity of aphasia therapy, impact on recovery. Stroke. 2003;34(4):987–93. doi: 10.1161/01.STR.0000062343.64383.D0 12649521

[3] Brady MC, Kelly H, Godwin J, Enderby P, Campbell P. Speech and language therapy for aphasia following stroke. Cochrane Database Syst Rev. 2016;2016(6):CD000425. doi: 10.1002/14651858.CD000425.pub4 27245310 PMC8078645

[4] Breitenstein C, Grewe T, Flöel A, Ziegler W, Springer L, Martus P, et al. Intensive speech and language therapy in patients with chronic aphasia after stroke: a randomised, open-label, blinded-endpoint, controlled trial in a health-care setting. Lancet. 2017;389(10078):1528–38. doi: 10.1016/S0140-6736(17)30067-3 28256356

[5] Cao Y, Huang X, Zhang B, Kranz GS, Zhang D, Li X, et al. Effects of virtual reality in post-stroke aphasia: a systematic review and meta-analysis. Neurol Sci. 2021;42(12):5249–59. doi: 10.1007/s10072-021-05202-5 33834356

[6] Lefaucheur J-P, Antal A, Ayache SS, Benninger DH, Brunelin J, Cogiamanian F, et al. Evidence-based guidelines on the therapeutic use of transcranial direct current stimulation (tDCS). Clin Neurophysiol. 2017;128(1):56–92. doi: 10.1016/j.clinph.2016.10.087 27866120

[7] Xu A-H, Sun Y-X. Research hotspots and effectiveness of repetitive transcranial magnetic stimulation in stroke rehabilitation. Neural Regen Res. 2020;15(11):2089–97. doi: 10.4103/1673-5374.282269 32394967 PMC7716019

[8] Harvey DY, Hamilton R. Noninvasive brain stimulation to augment language therapy for poststroke aphasia. Handb Clin Neurol. 2022;185:241–50. doi: 10.1016/B978-0-12-823384-9.00012-8 35078601

[9] Baker JM, Rorden C, Fridriksson J. Using transcranial direct-current stimulation to treat stroke patients with aphasia. Stroke. 2010;41(6):1229–36. doi: 10.1161/STROKEAHA.109.576785 20395612 PMC2876210

[10] Fridriksson J. Preservation and modulation of specific left hemisphere regions is vital for treated recovery from anomia in stroke. J Neurosci. 2010;30(35):11558–64. doi: 10.1523/JNEUROSCI.2227-10.2010 20810877 PMC2938788

[11] Richter M, Miltner WHR, Straube T. Association between therapy outcome and right-hemispheric activation in chronic aphasia. Brain. 2008;131(Pt 5):1391–401. doi: 10.1093/brain/awn043 18349055

[12] Kang EK, Kim YK, Sohn HM, Cohen LG, Paik N-J. Improved picture naming in aphasia patients treated with cathodal tDCS to inhibit the right Broca’s homologue area. Restor Neurol Neurosci. 2011;29(3):141–52. doi: 10.3233/RNN-2011-0587 21586821 PMC4886370

[13] You DS, Kim D-Y, Chun MH, Jung SE, Park SJ. Cathodal transcranial direct current stimulation of the right Wernicke’s area improves comprehension in subacute stroke patients. Brain Lang. 2011;119(1):1–5. doi: 10.1016/j.bandl.2011.05.002 21641021

[14] Anglade C, Thiel A, Ansaldo AI. The complementary role of the cerebral hemispheres in recovery from aphasia after stroke: a critical review of literature. Brain Inj. 2014;28(2):138–45. doi: 10.3109/02699052.2013.859734 24456053

[15] Datta A, Baker JM, Bikson M, Fridriksson J. Individualized model predicts brain current flow during transcranial direct-current stimulation treatment in responsive stroke patient. Brain Stimul. 2011;4(3):169–74. doi: 10.1016/j.brs.2010.11.001 21777878 PMC3142347

[16] Turkeltaub PE, Swears MK, D’Mello AM, Stoodley CJ. Cerebellar tDCS as a novel treatment for aphasia? Evidence from behavioral and resting-state functional connectivity data in healthy adults. Restor Neurol Neurosci. 2016;34(4):491–505. doi: 10.3233/RNN-150633 27232953 PMC5469248

[17] Xing S, Lacey EH, Skipper-Kallal LM, Jiang X, Harris-Love ML, Zeng J, et al. Right hemisphere grey matter structure and language outcomes in chronic left hemisphere stroke. Brain. 2016;139(Pt 1):227–41. doi: 10.1093/brain/awv323 26521078 PMC4990653

[18] Skipper-Kallal LM, Lacey EH, Xing S, Turkeltaub PE. Right hemisphere remapping of naming functions depends on lesion size and location in poststroke aphasia. Neural Plast. 2017;2017:8740353. doi: 10.1155/2017/8740353 28168061 PMC5266856

[19] Breining BL, Sebastian R. Neuromodulation in post-stroke aphasia treatment. Curr Phys Med Rehabil Rep. 2020;8(2):44–56. doi: 10.1007/s40141-020-00257-5 33344066 PMC7748105

[20] Marangolo P, Fiori V, Caltagirone C, Pisano F, Priori A. Transcranial cerebellar direct current stimulation enhances verb generation but not verb naming in poststroke aphasia. J Cogn Neurosci. 2018;30(2):188–99. doi: 10.1162/jocn_a_01201 29064340

[21] Sebastian R, Kim J, Brenowitz R, Tippett D, Desmond J, Celnik P. Cerebellar neuromodulation improves naming in post-stroke aphasia. Brain communications. 2020;2:fcaa179. 10.1093/braincomms/fcaa179 PMC7677607 33241212

[22] DeMarco AT, Dvorak E, Lacey E, Stoodley CJ, Turkeltaub PE. An exploratory study of cerebellar transcranial direct current stimulation in individuals with chronic stroke aphasia. Cogn Behav Neurol. 2021;34(2):96–106. doi: 10.1097/WNN.0000000000000270 34074864 PMC8186819

[23] Mariën P, Borgatti R. Language and the cerebellum. Handb Clin Neurol. 2018;154:181–202. doi: 10.1016/B978-0-444-63956-1.00011-4 29903439

[24] Vlasova RM, Panikratova YR, Pechenkova EV. Systematic review and meta-analysis of language symptoms due to cerebellar injury. Cerebellum. 2023;22(6):1274–86. doi: 10.1007/s12311-022-01482-5 36205825

[25] van Dun K, Bodranghien F, Manto M, Mariën P. Targeting the cerebellum by noninvasive neurostimulation: a review. Cerebellum. 2017;16(3):695–741. doi: 10.1007/s12311-016-0840-7 28032321

[26] van Dun K, Mitoma H, Manto M. Cerebellar cortex as a therapeutic target for neurostimulation. Cerebellum. 2018;17(6):777–87. doi: 10.1007/s12311-018-0976-8 30276522

[27] Celnik P. Understanding and modulating motor learning with cerebellar stimulation. Cerebellum. 2015;14(2):171–4. doi: 10.1007/s12311-014-0607-y 25283180 PMC4348328

[28] Gao Z, Davis C, Thomas AM, Economo MN, Abrego AM, Svoboda K, et al. A cortico-cerebellar loop for motor planning. Nature. 2018;563(7729):113–6. doi: 10.1038/s41586-018-0633-x 30333626 PMC6212318

[29] Wessel MJ, Hummel FC. Non-invasive cerebellar stimulation: a promising approach for stroke recovery?. Cerebellum. 2018;17(3):359–71. doi: 10.1007/s12311-017-0906-1 29243202

[30] Carta I, Chen CH, Schott AL, Dorizan S, Khodakhah K. Cerebellar modulation of the reward circuitry and social behavior. Science. 2019;363(6424):eaav0581. doi: 10.1126/science.aav0581 30655412 PMC6711161

[31] Pezzetta R, Gambarota F, Tarantino V, Devita M, Cattaneo Z, Arcara G, et al. A meta-analysis of non-invasive brain stimulation (NIBS) effects on cerebellar-associated cognitive processes. Neurosci Biobehav Rev. 2024;157:105509. doi: 10.1016/j.neubiorev.2023.105509 38101590

[32] Yao J, Liu X, Liu Q, Wang J, Ye N, Lu X, et al. Characteristics of non-linguistic cognitive impairment in post-stroke aphasia patients. Front Neurol. 2020;11:1038. doi: 10.3389/fneur.2020.01038 33117251 PMC7561418

[33] Higgins J, Barbieri E, Wang X, Mack J, Caplan D, Kiran S, et al. Reliability of BOLD signals in chronic stroke-induced aphasia. Eur J Neurosci. 2020;52(8):3963–78. doi: 10.1111/ejn.14739 32282965

[34] Schevenels K, Gerrits R, Lemmens R, De Smedt B, Zink I, Vandermosten M. Early white matter connectivity and plasticity in post stroke aphasia recovery. Neuroimage Clin. 2022;36:103271. doi: 10.1016/j.nicl.2022.103271 36510409 PMC9723316

[35] Spielmann K, van de Sandt-Koenderman WME, Heijenbrok-Kal MH, Ribbers GM. Transcranial direct current stimulation does not improve language outcome in subacute poststroke aphasia. Stroke. 2018;49(4):1018–20. doi: 10.1161/STROKEAHA.117.020197 29523651

[36] Woods AJ, Antal A, Bikson M, Boggio PS, Brunoni AR, Celnik P, et al. A technical guide to tDCS, and related non-invasive brain stimulation tools. Clin Neurophysiol. 2016;127(2):1031–48. doi: 10.1016/j.clinph.2015.11.012 26652115 PMC4747791

[37] Hurtado-Puerto AM, Nestor K, Eldaief M, Camprodon JA. Safety considerations for cerebellar theta burst stimulation. Clin Ther. 2020;42(7):1169-1190.e1. doi: 10.1016/j.clinthera.2020.06.001 32674957

[38] Fregni F, El-Hagrassy MM, Pacheco-Barrios K, Carvalho S, Leite J, Simis M, et al. Evidence-based guidelines and secondary meta-analysis for the use of transcranial direct current stimulation in neurological and psychiatric disorders. Int J Neuropsychopharmacol. 2021;24(4):256–313. doi: 10.1093/ijnp/pyaa051 32710772 PMC8059493

[39] Rossi S, Antal A, Bestmann S, Bikson M, Brewer C, Brockmöller J, et al. Safety and recommendations for TMS use in healthy subjects and patient populations, with updates on training, ethical and regulatory issues: Expert Guidelines. Clin Neurophysiol. 2021;132(1):269–306. doi: 10.1016/j.clinph.2020.10.003 33243615 PMC9094636

[40] Chan A-W, Tetzlaff JM, Altman DG, Laupacis A, Gøtzsche PC, Krleža-Jerić K, et al. SPIRIT 2013 statement: defining standard protocol items for clinical trials. Ann Intern Med. 2013;158(3):200–7. doi: 10.7326/0003-4819-158-3-201302050-00583 23295957 PMC5114123

[41] Schellinger PD, Bryan RN, Caplan LR, Detre JA, Edelman RR, Jaigobin C, et al. Evidence-based guideline: The role of diffusion and perfusion MRI for the diagnosis of acute ischemic stroke [RETIRED]: report of the Therapeutics and Technology Assessment Subcommittee of the American Academy of Neurology. Neurology. 2010;75(2):177–85. doi: 10.1212/WNL.0b013e3181e7c9dd 20625171 PMC2905927

[42] Risser AH, Spreen O. The western aphasia battery - Kertesz, A. J Clin Exp Neuropsychol. 1985; 7: 463–70.

[43] Faul F, Erdfelder E, Lang A-G, Buchner A. G*Power 3: a flexible statistical power analysis program for the social, behavioral, and biomedical sciences. Behav Res Methods. 2007;39(2):175–91. doi: 10.3758/bf03193146 17695343

[44] Faul F, Erdfelder E, Buchner A, Lang A-G. Statistical power analyses using G*Power 3.1: tests for correlation and regression analyses. Behav Res Methods. 2009;41(4):1149–60. doi: 10.3758/BRM.41.4.1149 19897823

[45] Rossi S, Hallett M, Rossini PM, Pascual-Leone A, Safety of TMS Consensus Group. Safety, ethical considerations, and application guidelines for the use of transcranial magnetic stimulation in clinical practice and research. Clin Neurophysiol. 2009;120(12):2008–39. doi: 10.1016/j.clinph.2009.08.016 19833552 PMC3260536

[46] Vasant DH, Michou E, Mistry S, Rothwell JC, Hamdy S. High-frequency focal repetitive cerebellar stimulation induces prolonged increases in human pharyngeal motor cortex excitability. J Physiol. 2015;593(22):4963–77. doi: 10.1113/JP270817 26316351 PMC4650408

[47] Katz N, Itzkovich M, Averbuch S, Elazar B. Loewenstein occupational therapy cognitive assessment (LOTCA) battery for brain-injured patients: reliability and validity. Am J Occup Ther. 1989;43:184–92. 2735378 10.5014/ajot.43.3.184

[48] Yu Z, Jiang S, Li J, Bi S, Li F, Xie T, et al. Clinical application of loewenstein occupational therapy cognitive assessment battery-second edition in evaluating of cognitive function of Chinese patients with post-stroke aphasia. Chin Med Sci J. 2013;28(3):167–71. doi: 10.1016/s1001-9294(13)60043-7 24074619

[49] Johnson EW. Visual analog scale (VAS). Am J Phys Med Rehabil. 2001;80(10):717. doi: 10.1097/00002060-200110000-00001 11562551

[50] Xue X-L, Wang T-F, Yu C-G. Estimation on the reliability and validity of the fatigue self-assessment scale. Zhongguo Zhong Xi Yi Jie He Za Zhi. 2008;28(6):550–4. 18655569

[51] Yao J, Liu X, Lu X, Xu C, Chen H, Zhang Y. Changes in white matter microstructure related to non-linguistic cognitive impairment in post-stroke aphasia. Neurol Res. 2020;42(8):640–8. doi: 10.1080/01616412.2020.1795578 32697169

[52] Liu Q, Zhang Y, Liu C, Chen Y, Zhang Y. Reduced cerebral blood flow and cognitive dysfunction following isolated cerebellar infarction: two case reports. J Int Med Res. 2024;52(3):3000605241235848. doi: 10.1177/03000605241235848 38513145 PMC10958817

[53] Hariton E, Locascio JJ. Randomised controlled trials - the gold standard for effectiveness research: study design: randomised controlled trials. BJOG. 2018;125(13):1716. doi: 10.1111/1471-0528.15199 29916205 PMC6235704

[54] Hordacre B, Austin D, Brown KE, Graetz L, Pareés I, De Trane S, et al. Evidence for a window of enhanced plasticity in the human motor cortex following ischemic stroke. Neurorehabil Neural Repair. 2021;35(4):307–20. doi: 10.1177/1545968321992330 33576318 PMC7610679

[55] Saur D, Lange R, Baumgaertner A, Schraknepper V, Willmes K, Rijntjes M, et al. Dynamics of language reorganization after stroke. Brain. 2006;129(Pt 6):1371–84. doi: 10.1093/brain/awl090 16638796

[56] Stockert A, Wawrzyniak M, Klingbeil J, Wrede K, Kümmerer D, Hartwigsen G, et al. Dynamics of language reorganization after left temporo-parietal and frontal stroke. Brain. 2020;143(3):844–61. doi: 10.1093/brain/awaa023 32068789

[57] Priori A, Hallett M, Rothwell JC. Repetitive transcranial magnetic stimulation or transcranial direct current stimulation?. Brain Stimul. 2009;2(4):241–5. doi: 10.1016/j.brs.2009.02.004 20633424

[58] Hardwick RM, Lesage E, Miall RC. Cerebellar transcranial magnetic stimulation: the role of coil geometry and tissue depth. Brain Stimul. 2014;7(5):643–9. doi: 10.1016/j.brs.2014.04.009 24924734 PMC4180011

[59] Sebastian R, Kim JH, Brenowitz R, Tippett DC, Desmond JE, Celnik PA. Cerebellar neuromodulation improves naming in post-stroke aphasia. Brain Communications. 2020;2:fcaa179. 10.1093/braincomms/fcaa179 PMC7677607 33241212

