# Supplementary material for: The application of cerebellar repetitive transcranial magnetic stimulation on aphasia after stroke: A randomized controlled trial protocol
Source: PLoS One. 2026 Aug 10;21(8):e0354837. doi: 10.1371/journal.pone.0354837 (PMC13456252; doi:10.1371/journal.pone.0354837)
Supplement: Supplementary file 1 [file pone.0354837.s002.pdf]

知情同意书（82572919）

研究方案名称（项目编号）：以小脑为靶点的经颅磁刺激技术在卒中后运动性失语患者语言康复中的作用及脑网络机制研究方案（82572919）

申办单位：首都医科大学附属北京天坛医院

CRO：无

版本号：*V4.0*

版本日期：2026.4.20

知情同意书

本文介绍的临床研究是由首都医科大学附属北京天坛医院发起的一项临床研究。作为研究人员，我们将向您介绍这个研究。参与本研究纯属自愿。

本文涵盖的部分内容由法规要求而定，并且为了保护参加研究的患者的权益，本文经首都医科大学附属北京天坛医院伦理委员会审核并同意。

1. 为什么要进行此项研究？

研究背景：卒中后失语（post-stroke aphasia, PSA）是指因急性脑血管疾病引起的言语障碍，表现为对语言符号的感知、理解、组织运用或表达等某一方面或多方面功能障碍。是卒中后严重的功能障碍之一。其中运动性失语(Broca's aphasia, BA）以口语表达障碍为最突出，主要表现为说话费力、不流利，语量稀少、呈电报式（主要是实质词）。既往研究发现应用经颅磁刺激（Transcranial Magnetic Stimulation, TMS） 治疗可促进失语症患者语言功能的恢复，且小脑具有独特的可塑性机制和与皮质区域的广泛连接，有望成为失语症康复的潜在目标。但小脑通过大脑-小脑环路在PSA 患者语言功能中的脑网络机制尚不清楚。

研究目的：阐明小脑在卒中后 BA 患者语言功能损害中的作用；明确以右侧小脑为靶点的 TMS 治疗对 BA 患者的语言康复效果。探讨小脑通过大脑-小脑环路调控语言功能的动态脑网络可塑性机制。

2. 多少人将参加这项研究？

首都医科大学附属北京天坛医院计划邀请 110 人参与本研究。

3. 研究选择哪些人参加？

我们将邀请符合以下要求的患者参加我们的试验:①年龄 18 岁至 80 周岁(包含 18 岁和 80 岁)，小学及以上文化程度；②母语为标准普通话，右利手，无左利手家族史；③病程介于 14 至 60 天，发病时以语言障碍为主；④首次发病，经 CT/MRI 证实为脑卒中，病变位于左侧大脑半球且病灶单一；⑤临床症状以语言功能障碍为主，且符合运动性失语；排除失语症合并严重非语言认知功能损害的患者；⑥能够配合完成神经心理学测评以及影像学检查；⑦签署知情同意书。

您是否可以参加研究，需要医生检查后最后决定。

4. 哪些人不宜参加研究？

如果您符合以下情况：①发病前已有语言功能障碍或合并其它原因导致的语言障碍；短暂性脑缺血发作病史；烟雾病，淀粉样血管变性等与认知功能障碍相关的病因引起的脑卒中；②存在潜在危险的既往病史，如癫痫、精神健康问题、视力听力问题、严重的脏器功能不全；③有开颅手术使或既往有癫痫发作；④合并严重构音障碍：⑤合并意识障碍格拉斯哥昏述评分 15 分；⑥影像学发现脑白质病变，微出血，血管周围间隙扩大，脑萎缩等脑小血管病变；⑦或本次发病后合并抑郁状态(医院版卒中后失语抑郁问卷(第 10 版)评分〉6 分)；⑧无法完成 TMS 治疗；⑨磁共振扫描过程中，头部移动明显(>3mm)，影响图像效果；那您将不属于本次研究范畴。

5. 参加这项研究会占用我多长时间？

这项研究计划将持续3年，会有专业人员定期与您取得联系，我们将通过电话以及到院检查等方式来收集您的相关疾病信息。

您可以在任何时间选择退出研究而不会丧失您本应获得的任何利益。然而，如果在研究途中您决定退出本研究，我们鼓励您先和您的医生商议。考虑到您的安全性问题，有可能在退出后，会进行一次相关检查。

6. 该研究是怎样进行的？

如果您愿意参加本项研究，您的医生会对您的病史进行了解，询问既往和目前的治疗用药情况，进行初步的语言功能测评，进一步确认您是否适合参加本研究。

本研究将比较以右侧小脑为靶点TMS组和假刺激组。由计算机对所有病人随机分组，您将有50%的机会被分到以右侧小脑为靶点TMS组，50%的机会被分到假刺激组。我们会在您接受治疗的12周内定期通过一系列检查和认知/语言功能测评来评估您语言功能的恢复情况，并会在治疗结束后1个月、3个月、6个月内对您进行随访。在上述治疗/检查中，经颅磁刺激治疗是试验性的治疗（即，如果您不参加本研究，就不需要接受该治疗）。

7. 参加研究我需要遵守哪些义务？

研究期间，您需要做以下事情：

1) 有义务如实提供病史和“此前参加临床试验的情况”，以及声明无精神障碍病史；

2) 配合医生进行语言/认知功能训练；

3) 在研究期间，您需要回到医院做至少 3 次的临床回访（训练 1 个月、3 个月、6 个月后，主要包含语言/认知量表测评和 MRI 检查）；

4) 在研究结束时或您决定退出研究时，您需要告知我们原因。

研究结束后：您需要告知我们参与本次康复训练/磁刺激治疗后的语言功能的主观恢复程度。

8. 参加研究涉及的费用有哪些？

（1）需要您承担的费用包括：在首都医科大学附属北京天坛医院或医联体医院的住院费用不在免费范围之内。如果您同时合并其他疾病所需的治疗和检查，以及因治疗无效而改用其他治疗的费用，也不在免费的范围之内。

（2）不需要您承担的费用包括：因本次研究而做的语言+认知功能评估、头 MRI 检查、TMS治疗是免费的。为了补偿您参加本研究可能给您带来的不便，本研究将支付您参加本项研究期间随访时的交通费100元/人次。

9. 参加研究对我的疾病治疗有哪些好处？

参加本项研究，您的病情有可能会、也可能不会获得改善，从本项研究中得到的信息将有助于您获得具有针对性的卒中后失语症相关的语言康复治疗意见，并有助于确定哪种治疗方法可以更安全有效地治疗与您患有相似病情的其他患者。

10. 我是否有其他的治疗选择？

参加本研究可能改善或不能改善您的健康状况。如果您不参加本研究，您可以选择如下治疗：

（1）在我院康复科进行常规语言康复治疗；

（2）在外院康复科进行语言康复治疗；

（3）居家自行康复锻炼，该方式由于没有专业的康复医生指导，进行不当可能会错过语言康复的黄金治疗期；

（4）居家静养，不进行康复治疗，该方式对于患者的语言康复的益处十分有限。

11. 参加研究可能有哪些风险？

经颅磁刺激治疗可能引起的不良反应如下：局部的烧灼感、头痛、耳鸣、焦虑、抑郁、癫痫等。此外任何治疗都有可能出现无效的情况，以及因治疗无效或合并其他疾病而导致病情继续发展。如果您的健康确因参加这项研究而发生与研究相关的损害，请立即通知研究医生，他们将负责对您采取适当的治疗措施。申办者北京天坛医院将按国家有关规定承担治疗费用及对您给予相应的经济补偿。

即使您已经签署这份知情同意书，您仍然保留您所有的合法权利。

12. 我可以自愿选择参加研究和中途退出研究吗？

是否参加本项研究是完全自愿的，您可以拒绝参加此项研究，或在研究过程中的任何时间退出本研究，不需要任何理由。该决定不会影响医生对您的治疗，其医疗待遇与权益也不会受到影响。

出于对您的最大利益考虑，医生或研究者可能在研究过程中随时中止您继续参加本研究。

如果您因为任何原因从本研究中退出，如果医生认为临床需要，您可能被要求进行实验室检查和体格检查，这对保护您的健康是有利的。

13. 如果有了与研究相关的新信息，会怎么样？

如有任何新的可能影响您继续参加本研究意愿的相关信息时，研究者会及时通知您，并会和您讨论是否适合继续参加本研究。

14. 参加该研究将如何影响我的生活？

您可能会觉得这些访视和检查会带来不便，并且需要特殊的安排。此外，一些检查还会使您感觉到不舒服。如果您有关于研究中检查和步骤的任何疑问可以向研究医生咨询。

在整个研究期间您不能再参加其他任何有关药物、医疗器械和其他康复手段的临床研究。

15. 我的个人信息是保密的吗？

您的医疗记录将保存在医院，研究者、研究主管部门人员、伦理委员会、监查员、稽查员和上级管理部门检查人员可以查阅受试者的原始医学记录，以核实临床试验的过程和数据。上述人员对您的个人信息有保密责任，违规泄露会被处罚。任何与您相关身份鉴别记录的保密事宜，均不公开使用。如果发布临床试验结果，您的身份信息仍将保密。我们将在法律允许的范围内，尽一切努力保护您个人医疗资料的隐私。您的姓名不会在任何报告中体现。

16. 相关咨询

如果您有与本研究相关的任何问题，请联系*刘医生*，固定电话及手机*13691571256*。

如果您有与自身权益相关的任何问题，或者您想反映参与本研究过程中的不满和疑问，请联系北京天坛医院国家临床试验机构办公室（注册研究），电话：010-59975178，或天坛医院国家医学中心（横向课题），电话 010-59976165，或天坛医院科技处（纵向课题），电话 010-59975825/5827/5832/6171。

天坛医院伦理委员会办公室，联系电话：010-59978555/5692/6269。邮箱：ttyyirb@163.com。

受试者同意声明

同意参加“以小脑为靶点的经颅磁刺激技术在卒中后运动性失语患者语言康复中的作用及脑网络机制研究”的临床研究

在此签字，意味着：

1. 我已读过本知情同意书，且研究人员已经向我解释了该研究。
2. 我已经讨论并询问了有关本研究的相关问题，这些问题的解答令我满意。
3. 我知道如果出现与研究相关的损害，我能够从申办者/资质者（二选一保留）获得赔偿。
4. 我有充足的时间做出决定。
5. 我是自愿同意参加本文所介绍的临床研究。
6. 我已经获知在研究中我该咨询的研究人员名单。
7. 如本知情同意书所介绍，我同意与研究相关的人员和监管部门人员，必要时能够接触到我的医疗和个人信息。
8. **首都医科大学附属北京天坛医院作为教学医院，肩负有治疗、教学与科研的任务，目的是在治疗疾病的基础上，培养医学生成为合格的医师，同时利用现有资源，不断提高对疾病的诊疗能力，更好地服务于患者。您是否同意您的研究数据及样本用于医院将来的医学研究（如类似疾病的分析研究等），这完全是出于自愿，您在本研究项目中或其他任何医疗活动中的待遇与权益不会因此而受到影响。**

在严格保护个人身份信息的前提下，本人/监护人 □同意 □不同意（请在方框内用“ √ ”代表选择）

本研究的研究数据与样本在伦理委员会批准的情况下用于其他回顾性研究。

您有权在任何时间撤回上述同意，撤回请联系签署本知情同意书的研究者。

受试者签名：______________　　日期：______________

姓名正楷：______________　　联系电话：

监护人签名（如有）：______________　　日期：______________

监护人姓名正楷：______________　　联系电话：

监护人与患者关系：

公正见证人声明：

我在知情过程中全程在场，知情同意书中的内容以及其他文字性资料中的内容准确解释给受试者或监护人，受试者或监护人完全理解了内容含义，他们表示同意参加试验。

公正见证人签名（如有）：______________　　日期：______________

公正见证人姓名正楷：______________　　联系电话：

研究者签名：______________　　日期：______________

研究者姓名正楷：______________　　联系电话：
