# Supplementary material for: The application of cerebellar repetitive transcranial magnetic stimulation on aphasia after stroke: A randomized controlled trial protocol
Source: PLoS One. 2026 Aug 10;21(8):e0354837. doi: 10.1371/journal.pone.0354837 (PMC13456252; doi:10.1371/journal.pone.0354837)
Supplement: Supplementary file 2 [file pone.0354837.s004.docx]

**Study Protocol (English Version)**

Basic Information

Project Source & Grant No.: National Natural Science Foundation of China (No. 82572919)

Study Protocol: Effects and Cerebro-Cerebellar Network Mechanisms of Cerebellum-Targeted Transcranial Magnetic Stimulation on Language Rehabilitation in Patients with Post-Stroke Motor Aphasia

Full Project Name: Effects and Cerebro-Cerebellar Network Mechanisms of Cerebellum-Targeted Transcranial Magnetic Stimulation on Language Rehabilitation in Patients with Post-Stroke Motor Aphasia

Principal Investigator / Department: Zhang Yumei / Department of Rehabilitation Medicine

Entrusting Institution: Beijing Tiantan Hospital, Capital Medical University

Undertaking Institution: Beijing Tiantan Hospital, Capital Medical University

Lead Institution: Beijing Tiantan Hospital, Capital Medical University

Study Period: January 2026 – December 2029

Version Number: V1.0

Version Date: January 8, 2026

Protocol Summary

Item Content

Project Title: Effects and Cerebro-Cerebellar Network Mechanisms of Cerebellum-Targeted Transcranial Magnetic Stimulation on Language Rehabilitation in Patients with Post-Stroke Motor Aphasia

Study Objectives:

1. To clarify the role of the cerebellum in language impairment of patients with post-stroke motor aphasia.

2. To confirm the therapeutic effect of right cerebellum-targeted transcranial magnetic stimulation (TMS) on language rehabilitation in post-stroke aphasia patients.

3. To explore the dynamic brain network plasticity mechanism through which the cerebellum regulates language function via the cerebro-cerebellar circuit.

Study Design Prospective randomized controlled study

Total Number of Subjects 1. Part 1 (observation group): 44 patients with post-stroke motor aphasia and 44 healthy controls.

2. Part 2 (intervention group): 60 patients with post-stroke motor aphasia.

Total: 110 aphasic patients and 44 healthy controls.

Inclusion Criteria for Subjects Aged 18 to 80 years old (inclusive); primary school education or above;

Native Mandarin speaker, right-handed, no family history of left-handedness;

Disease course ranging from 14 to 60 days, with language disorder as the primary onset manifestation;

Patients with TMS contraindications will be excluded in Part 2;

First-ever stroke confirmed by CT/MRI, with a single lesion located in the left cerebral hemisphere;

Diagnosed with Broca’s aphasia (BA) via Western Aphasia Battery; cognitive function evaluated by Loewenstein Occupational Therapy Cognitive Assessment; patients with severe non-verbal cognitive impairment accompanied by aphasia will be excluded;

Able to complete neuropsychological assessments and imaging examinations;

Voluntarily sign the written informed consent form.

Exclusion Criteria for Subjects 1. Pre-existing language disorders before this stroke, or language dysfunction caused by other diseases; history of transient ischemic attack; stroke induced by moyamoya disease, cerebral amyloid angiopathy and other diseases related to cognitive impairment.

2. History of schizophrenia and other mental illnesses; patients with TMS contraindications will be excluded in Part 2.

3. Combined severe cardiac, hepatic or renal insufficiency; combined visual field defects or peripheral hearing impairment.

4. History of craniotomy or previous epileptic seizures.

5. Combined severe dysarthria; impaired consciousness (Glasgow Coma Scale score less than 15).

6. Brain small vessel lesions detected by imaging, including leukoaraiosis, cerebral microbleeds, enlarged perivascular spaces and cerebral atrophy.

7. Combined depressive state after stroke (Hospital Stroke Aphasia Depression Questionnaire, 10th edition, score > 6).

8. Recurrent stroke or new intracranial space-occupying lesions during the study.

9. Refusal to sign the informed consent form.

10. Inability to complete TMS treatment.

11. Obvious head movement (>3 mm) during MRI scanning, affecting image quality.

12. Failure to complete follow-up visits.

13. Voluntary withdrawal from the study by the patient.

Treatment Protocol 1. Baseline information collection (at enrollment)

① Demographic information: age, gender, handedness, education level, living status.

② Stroke risk factors: hypertension, diabetes, hyperlipidemia, coronary heart disease, smoking, body mass index, blood routine, liver function, renal function, homocysteine level.

③ Cerebrovascular disease-related information: stroke onset date, stroke type, lesion location.

2. Language, cognitive and brain imaging assessments (within 7 days of enrollment)

(1) Scale assessments

① Edinburgh Handedness Inventory

② Language function assessments: Western Aphasia Battery, Verbal Fluency Test, Phonological Test, Lexical Test, Grammar Test (Sentence Production), Boston Diagnostic Aphasia Examination

③ Instrumental Activities of Daily Living Scale, Stroke and Aphasia Quality of Life Scale

(2) Brain imaging assessment: Head MRI (T1WI, T2WI, T2-FLAIR, SWI, DTI, rs-fMRI)

3. Rehabilitation treatment

After enrollment, subjects will be randomly divided into two groups:

Group 1: Conventional language rehabilitation training + active stimulation targeting the right cerebellum

Group 2: Conventional language rehabilitation training + sham stimulation targeting the right cerebellum

Treatment schedule: Language rehabilitation training twice a day in the morning and afternoon, including 30 minutes of conventional language rehabilitation training and about 40 seconds of cTBS stimulation (total stimulation time approximately 1 minute), with a total session duration of 31 minutes. Training will be conducted 5 days a week for 4 consecutive weeks.

(1) Conventional language rehabilitation training: One-on-one computer-assisted individualized language training will be provided to patients by speech therapists from Beijing Tiantan Hospital, targeting verbal fluency, phonology, lexicon, grammar, auditory comprehension and oral expression. The training scheme adopts the research achievements of our previous National Natural Science Foundation project (No. 81972144), and training will be carried out based on patients’ residual language functions.

(2) TMS treatment protocol: Model YRD CCY-I magnetic stimulator with figure-8 coil will be used for cTBS treatment in BA patients.

Stimulation parameters: Intra-burst frequency 50 Hz, inter-burst frequency 5 Hz, stimulation intensity 80% of the motor threshold of the unaffected cerebral hemisphere (motor threshold refers to the minimum magnetic field intensity that can induce at least 5 hand twitches in 10 stimulations of the hand area of the motor cortex), stimulation duration 40 seconds, total pulses 600. The sham stimulation group will receive pseudo-stimulation pulses with the same parameters.

Scalp positioning follows the electrode positioning standard of the International Federation of Societies for Electroencephalography and Clinical Neurophysiology:

① Target of the active stimulation group: right cerebellum, located 1 cm below the inion and 3 cm to the right of the midline (The Journal of Physiology, 2015, 593(22):4963-4977), with the coil tangent to the scalp.

② The sham stimulation group adopts the same target as the active group, and the coil is tangent to the scalp.

Efficacy Evaluation Indicators 1. Primary evaluation indicators

① The role of the cerebellum in language impairment of post-stroke BA patients.

② Dynamic brain network plasticity mechanism by which the cerebellum regulates language function through the cerebro-cerebellar circuit.

2. Secondary evaluation indicators

① To evaluate the effectiveness of different rehabilitation training schemes.

② To explore factors affecting language function recovery during follow-up, including education level and stroke severity.

Safety evaluation indicators

After each TMS treatment session, questionnaires will be used to evaluate whether TMS treatment causes adverse reactions such as local burning sensation, head discomfort or pain, facial and mandibular muscle twitching, auditory hypersensitivity, tinnitus, syncope, mood swings, inattention and epileptic seizures in all patients. Timely symptomatic treatment will be provided if any safety problems occur in patients.

Statistical Methods Intergroup comparison of baseline indicators

SPSS 25.0 statistical software will be used to establish a database and conduct statistical analysis. Normality test will be performed on data; measurement data (such as fractional anisotropy, nodal degree, etc.) will be expressed as mean ± standard deviation or median (lower quartile, upper quartile), and intergroup comparisons will adopt t-test, Wilcoxon test or Kruskal-Wallis rank-sum test. Count data will be expressed as rate, and intergroup comparisons will adopt chi-square test, Fisher’s exact test or Wilcoxon rank-sum test. P < 0.05 is considered statistically significant.

Efficacy analysis

1. Clinical indicators: Based on the principle of Intention-To-Treat (ITT) analysis, repeated measures analysis of variance or Wilcoxon rank-sum test will be used to evaluate the improvement of language function of subjects at different time points before and after rehabilitation treatment in each group, and to compare the rehabilitation efficacy between the right cerebellum active TMS group and the right cerebellum sham stimulation group. P < 0.05 is considered statistically significant.

2. Imaging indicators: Pearson correlation analysis, dynamic functional connectivity density analysis and cluster analysis will be conducted between language function scores of BA groups (including each subgroup before and after treatment) and FA value, nodal degree, ALFF value, Functional Connectivity Density (FCD), and comparative analysis with the HC group will be performed to evaluate the role of the cerebellum in language impairment and neuroplasticity mechanism. Voxels with FDR-corrected q < 0.05 are defined as statistically significant.

Study Duration January 2026 – December 2029

1. Research Background

Stroke is one of the major diseases endangering national health in China. Large-sample national multicenter studies conducted by Beijing Tiantan Hospital have verified that stroke is characterized by high incidence, high disability rate, high mortality and high recurrence rate. Most stroke patients suffer from residual dysfunction, among which aphasia is a common sequela with an incidence rate of 21–42%. Improving the language function and communication ability of aphasic patients is an urgent problem to be solved in the field of stroke research.

Previous studies have found that Transcranial Magnetic Stimulation (TMS) therapy can promote the recovery of language function in aphasic patients. Based on meta-analyses and domestic and foreign expert consensus and guidelines, the recommended TMS targets for post-stroke aphasia (PSA) patients are mainly peri-lesional areas and contralesional mirror areas, but both targets have certain limitations. At present, the regulatory effect of the cerebellum on language function has attracted widespread attention. Studies have found that there are extensive neural circuits between the cerebellum and the frontal cortex, and the cerebellum has functional lateralization: the left cerebellum plays an important role in cognitive control through more connections with the prefrontal lobe, parietal lobe and subcortical cerebral regions, while the right cerebellum is more involved in language control. Therefore, the cerebellum has a unique plasticity mechanism and extensive connections with cortical regions, and is expected to become a potential target for aphasia rehabilitation.

Preliminary research of our group has found that aphasic patients have extensive changes in fiber connections, including alterations in cerebro-cerebellar fiber tracts. Therefore, we hypothesize that the cerebellum plays a role in language impairment of PSA patients through the cerebro-cerebellar circuit, especially the right cerebellum, which is expected to be a potential target for TMS treatment of aphasia. However, existing studies on cerebellar TMS intervention for PSA have small sample sizes and fail to observe efficacy at different time points after treatment due to limited follow-up conditions. Therefore, there is no unified conclusion on the rehabilitation efficacy of cerebellum-targeted TMS on language function and its underlying brain network plasticity mechanism in PSA patients.

Therefore, relevant basic and clinical research is urgently needed to explore the brain network mechanism of the cerebellum regulating language function in PSA patients via the cerebro-cerebellar circuit. This randomized controlled trial will dynamically observe the recovery of language function and plastic changes of brain networks at different time points after right cerebellum-targeted TMS treatment, further explore the connection between the cerebellum and language networks, and tap the potential of the cerebellum as a TMS stimulation target to improve the language prognosis of aphasic patients.

Objectives

2.1 Primary Objectives

1. To clarify the role of the cerebellum in language impairment of post-stroke BA patients.

2. To confirm the language rehabilitation efficacy of right cerebellum-targeted TMS therapy in BA patients.

3. To explore the dynamic brain network plasticity mechanism by which the cerebellum regulates language function through the cerebro-cerebellar circuit.

2.2 Secondary Objectives

1. To clarify the involvement of the cerebellum in language impairment of post-stroke BA patients, focusing on changes in cerebello-frontal functional connectivity strength.

2. To explore the rehabilitation efficacy and brain network plasticity mechanism of right cerebellum-targeted TMS therapy for BA patients.

3. Study Design, Principles and Trial Procedures

3.1 Study Design

This study consists of two parts. Part 1 enrolls post-stroke BA patients as the observation group to clarify the brain network mechanism of cerebellar involvement in language impairment of patients with post-stroke motor aphasia (Broca’s aphasia, BA). Part 2 enrolls post-stroke BA patients receiving rehabilitation training to clarify the rehabilitation efficacy and brain network plasticity mechanism of cerebellum-targeted TMS therapy. All subjects will receive TMS targeting the right cerebellum and be randomly divided into active stimulation group and sham stimulation group via computer randomization.

3.2 Sample Size and Research Plan

Part 1 Observation Study

To clarify the brain network mechanism of cerebellar involvement in language impairment of post-stroke BA patients, BA patients will be recruited as the observation group, and healthy subjects matched for age, gender, handedness and education level will be recruited as the HC group.

Statistical parameters: α=0.05, Z(1-α/2)=1.96, allowable error δ=0.1, p represents the specificity of the designed language tasks for BA patients. Combined with the number of subjects recruited in previous linguistic studies, 40 BA patients are initially planned. Considering loss to follow-up and subject dropout, a 10% sample size expansion will be added. Therefore, 44 BA patients and 44 healthy controls will be enrolled in Part 1.

Part 2 Randomized Controlled Intervention Study

To confirm the rehabilitation efficacy and brain network plasticity mechanism of cerebellum-targeted TMS therapy for BA patients, post-stroke BA patients will be enrolled and randomly divided into active stimulation group and sham stimulation group with equal sample sizes via computer randomization.

Adopting parallel-group design with equal sample size in two groups and two-sided difference test: α=0.05, β=0.20, p1 and p2 represent the language improvement rates of the right cerebellum TMS group and sham stimulation group at 3 months after stimulation respectively. Combined with previous linguistic research recruitment data, 60 BA patients are initially planned (30 in the right cerebellum TMS group and 30 in the sham stimulation group). Considering loss to follow-up and dropout, a 10% sample expansion will be added, so 66 BA patients will be enrolled in Part 2.

Total sample size of this study: 110 BA patients (44 in observation group + 66 in intervention group) and 44 healthy controls.

Study period: January 2026 – December 2029.

3.3 Subject Eligibility Criteria

Inclusion Criteria for BA Patients

1. Aged 18 to 80 years old (inclusive); primary school education or above.

2. Native Mandarin speaker, right-handed, no family history of left-handedness.

3. Disease course 14 to 60 days, with language disorder as the main onset manifestation.

4. Patients with TMS contraindications will be excluded in Part 2.

5. First-ever stroke confirmed by CT/MRI, with a single lesion located in the left cerebral hemisphere.

6. Diagnosed with BA via Western Aphasia Battery; cognitive function evaluated by Loewenstein Occupational Therapy Cognitive Assessment to exclude patients with severe non-verbal cognitive impairment accompanied by aphasia.

7. Able to complete neuropsychological assessments and imaging examinations.

8. Voluntarily sign the written informed consent form.

Exclusion Criteria for BA Patients

1. Pre-existing language disorders before this stroke, or language dysfunction caused by other etiologies; history of transient ischemic attack; stroke induced by moyamoya disease, cerebral amyloid angiopathy and other diseases related to cognitive impairment.

2. History of schizophrenia and other mental illnesses; patients with TMS contraindications will be excluded in Part 2.

3. Combined severe cardiac, hepatic or renal insufficiency.

4. Combined visual field defects or peripheral hearing impairment.

5. History of craniotomy or previous epileptic seizures; combined severe dysarthria; impaired consciousness (Glasgow Coma Scale score less than 15).

6. Brain small vessel lesions detected by imaging, including leukoaraiosis, cerebral microbleeds, enlarged perivascular spaces and cerebral atrophy.

7. Combined depressive state after stroke (Hospital Stroke Aphasia Depression Questionnaire, 10th edition, score > 6).

8. Recurrent stroke or new intracranial space-occupying lesions during the study.

9. Refusal to sign the informed consent form.

10. Inability to complete TMS treatment or follow-up visits.

Inclusion Criteria for Healthy Controls (HC)

1. Primary school education or above.

2. No history of cerebrovascular disease, traumatic brain injury, epilepsy, dementia or other neurological diseases.

3. Normal cardiac, pulmonary, hepatic and renal function.

4. No visual field defects or peripheral hearing impairment.

5. Normal language function confirmed by Western Aphasia Battery (Aphasia Quotient > 93.8) and normal cognitive function confirmed by Montreal Cognitive Assessment (total score ≥ 26).

6. No MRI contraindications; normal brain MRI results.

7. Voluntarily sign the written informed consent form.

Exclusion Criteria for Healthy Controls

1. History of stroke, intracranial space-occupying lesions and other brain diseases.

2. Confirmed history of schizophrenia and other mental illnesses, or combined depressive state.

3.4 Research Methods

Part 1 Observational Study

Behavioral and imaging assessments will be conducted on post-stroke BA patients to explore the role of the cerebellum and its brain network mechanism in language impairment of BA patients, verify the lateralization of right cerebellum in regulating language function, and lay a theoretical foundation for selecting the right cerebellum as the TMS stimulation target.

Recruitment of subjects → Neuropsychological assessment → Imaging data collection → Construction of behavioral-imaging parameter mapping

Linguistic assessments: VFT, WAB, Phonological Test, Lexical Test, Grammar Test, BDAE, IADL, SAQOL-39g

MRI sequences: T1WI, T2WI, T2-FLAIR, SWI, DTI, rs-fMRI

Analytical methods: TBSS, ALFF, FCD/dFCD, graph theory analysis

Research endpoints: Identify linguistic characteristics and brain structural and functional differences between BA patients and healthy controls; clarify the brain network mechanism of the cerebellum in the occurrence and development of BA.

Part 2 Randomized Controlled Clinical Study

Post-stroke BA patients will receive TMS therapy targeting the right cerebellum, and longitudinal observations will be conducted to explore the brain network plasticity mechanism of cerebellar participation in language function recovery.

3.4.1 Neuropsychological Assessment

• Part 1: BA patients and healthy controls will receive neuropsychological assessments at baseline (within ±7 days before treatment).

• Part 2: BA patients will receive neuropsychological assessments at four time points after stroke: baseline (within ±7 days before treatment), 2 weeks after treatment (±3 days), 1 month after treatment (±7 days), and 3 months after treatment (±14 days). Corresponding language tasks will be designed at different stages after stroke according to the evolution characteristics of language function, with differentiated task settings to clarify the characteristics of language impairment in BA patients at different stages and eliminate the interference of memory of language tasks on results.

1. Oral production function assessment

(1) Verbal Fluency Test (VFT): Subjects are required to spontaneously state a certain number of words according to rules within a fixed time without repetition (such as words starting with a certain initial consonant or names of animals), to evaluate semantic fluency, phonemic fluency and verb fluency.

(2) Western Aphasia Battery (WAB): The spontaneous speech subtest of WAB will be used to evaluate BA patients and calculate fluency scores.

(3) Multi-level oral production tasks

Phonological Test: Adopt the Chinese Articulation Test word list compiled by Huang Zhaoming and Han Zhijuan. The test material includes 50 monosyllabic words covering 21 initials, 13 finals and 4 tones. Phonological contrast, minimal phoneme contrast and phoneme acquisition will be analyzed to evaluate oral production at the phonological level.

Lexical Test: Adopt the lexical test corpus from Beijing Normal University. Subjects are presented with a series of black-and-white pictures for naming, including object naming and action naming. Boston Naming Test will be used to evaluate naming ability of BA patients.

Grammar Test (Sentence Production): This test includes sentence production and sentence comprehension. The sentence production subtest jointly developed by Beijing Language and Culture University and Northwestern University will be used to evaluate verb and verb argument structure production disorders. The sentence-picture matching subtest of Chinese Standard Aphasia Examination will be used for sentence comprehension evaluation.

2. Auditory comprehension and oral expression assessment

The subtests of WAB will be adopted: auditory comprehension subtest evaluates oral comprehension ability; repetition subtest evaluates repetition ability; reading and writing subtests evaluate reading and writing ability respectively.

3. Aphasia severity assessment

Boston Diagnostic Aphasia Examination (BDAE) will be used to grade the severity of aphasia.

4. Instrumental activities of daily living and quality of life assessment

Instrumental Activities of Daily Living Scale (IADL) and Stroke and Aphasia Quality of Life Scale (SAQOL-39g) will be used to evaluate daily living ability and quality of life of BA patients.

3.4.2 Imaging Data Collection

All MRI scans will be performed on a GE Signa HDxt 3.0T magnetic resonance scanner with an 8-channel head coil. Healthy controls will receive one scan session; BA patients will receive MRI scans at three time points: baseline (within ±7 days before treatment), 2 weeks after treatment (±3 days), and 1 month after treatment (±7 days). Rubber earplugs will be used to reduce noise during scanning, and foam pads will fix the head position to reduce motion artifacts. Subjects are required to keep eyes closed and awake during resting-state fMRI scanning.

MRI scan sequence parameters

Scan Sequence TR (ms) TE (ms) FOV (mm²) Slice Thickness (mm) Slice Gap (mm) Number of Slices Matrix Size Notes

T1WI 2500 4.37 256×256 0.8 0 208 256×256 —

T2WI 4500 84 220×220 1 0.5 28 256×256 —

T2-FLAIR 7800 91 199×220 5 5 24 256×256 —

SWI 27 20 230×230 2.6 0 48 256×256 —

DTI 13000 69.3 250×250 3.5 0 36 128×128 30 diffusion directions, b=0 and 1500 s/mm²

rs-fMRI 650 27 200×200 2 0.7 72 100×100 —

3.4.3 Rehabilitation Training

BA patients enrolled in Part 2 will be randomly divided into two groups for rehabilitation training:

Group 1: Conventional language rehabilitation training + active stimulation targeting the right cerebellum

Group 2: Conventional language rehabilitation training + sham stimulation targeting the right cerebellum

Training schedule: Language rehabilitation training twice a day in the morning and afternoon, 5 days a week for 4 consecutive weeks. Each session includes 30 minutes of conventional language rehabilitation training and 40 seconds of cTBS stimulation, with a total duration of 31 minutes.

(1) Conventional language rehabilitation training: One-on-one computer-assisted individualized language training will be provided by speech therapists from Beijing Tiantan Hospital, targeting verbal fluency, phonology, lexicon, grammar, auditory comprehension and oral expression. The training scheme adopts the research achievements of our previous National Natural Science Foundation project (No. 81972144), and training will be carried out based on patients’ residual language functions.

(2) TMS treatment protocol: Model YRD CCY-I magnetic stimulator with figure-8 coil will be used for cTBS treatment in BA patients. Stimulation parameters: Intra-burst frequency 50 Hz, inter-burst frequency 5 Hz, stimulation intensity 80% of the motor threshold of the unaffected cerebral hemisphere (motor threshold refers to the minimum magnetic field intensity that can induce at least 5 hand twitches in 10 stimulations of the hand area of the motor cortex), stimulation duration 40 seconds, total pulses 600. The sham stimulation group will receive pseudo-stimulation pulses with the same parameters.

Scalp positioning follows the electrode positioning standard of the International Federation of Societies for Electroencephalography and Clinical Neurophysiology:

① Target of the active stimulation group: right cerebellum, located 1 cm below the inion and 3 cm to the right of the midline (The Journal of Physiology, 2015, 593(22):4963-4977), with the coil tangent to the scalp.

② The sham stimulation group adopts the same target as the active group, and the coil is tangent to the scalp.

4. Concomitant Medications

No investigational drugs will be used in this study, and patients’ oral medications for underlying diseases will not be restricted.

5. Observation Indicators and Examination Timelines

1. Subjects meeting inclusion criteria will receive language and daily function assessments within 2 weeks after stroke onset, including Western Aphasia Battery, Phonological Test, Edinburgh Handedness Inventory, Lexical Test, Grammar Test, Verbal Fluency Test and Instrumental Activities of Daily Living Scale.

2. MRI data will be collected within 2 weeks after stroke onset using Siemens 3.0T magnetic resonance scanner at Beijing Tiantan Hospital, including T1WI, T2WI, 3D-FLAIR, rs-fMRI and DTI sequences.

3. Follow-up assessments: Behavioral data of language function will be collected within 3 days after rehabilitation training, 1 month after training (±7 days), and 3 months after training (±14 days). Imaging data will be collected within 3 days after rehabilitation training and 1 month after training (±7 days).

6. Follow-up Schedule

Behavioral assessments (language and cognitive function): Baseline (within ±7 days before treatment), 2 weeks after treatment (±3 days), 1 month after treatment (±7 days), 3 months after treatment (±14 days).

Imaging scans: Baseline (within ±7 days before treatment), 2 weeks after treatment (±3 days), 1 month after treatment (±7 days).

7. Clinical Evaluation

7.1 Efficacy Evaluation

Based on the principle of Intention-To-Treat (ITT) analysis, repeated measures analysis of variance or Wilcoxon rank-sum test will be used to evaluate the improvement of language function of subjects at different time points before and after rehabilitation treatment in each group, and to compare the rehabilitation efficacy between the right cerebellum active TMS group and the right cerebellum sham stimulation group. P < 0.05 is considered statistically significant.

7.2 Safety Evaluation

After each TMS treatment session, questionnaires will be used to evaluate whether TMS treatment causes adverse reactions such as local burning sensation, head discomfort or pain, facial and mandibular muscle twitching, auditory hypersensitivity, tinnitus, syncope, mood swings, inattention and epileptic seizures in all patients. Timely symptomatic treatment will be provided if any adverse events occur.

8. Known and Potential Risks, Benefits and Risk Disposal Plans

This study involves TMS treatment and language/cognitive rehabilitation training. Potential adverse reactions of TMS include local burning sensation, headache, tinnitus, anxiety, depression and epilepsy. No adverse reactions are associated with language/cognitive rehabilitation training. Other potential safety events include falls and recurrent stroke. All symptoms occurring during TMS treatment are defined as adverse events, and epileptic seizures are defined as serious adverse events.

Once an adverse event occurs, the patient and their family members will be informed immediately, brain imaging will be completed to rule out possible progression of primary disease, all details of the event will be recorded, and an internal research team meeting will be held within 3 working days to decide whether to terminate the subject’s participation in the study.

Once a serious adverse event occurs, medical intervention will be taken immediately, brain imaging will be completed, family members will be notified, the serious adverse event will be fully recorded, and an internal research team meeting will be held within 24 hours to terminate the subject’s participation in the study.

9. Quality Control and Quality Assurance

All researchers shall strictly follow the trial protocol, Good Clinical Practice (GCP) guidelines and relevant laws and regulations when conducting clinical research. All clinical operations shall comply with the standardized procedures specified in the protocol. Researchers shall provide reliable data and all information required by the trial protocol, and ensure that the monitoring team can directly access original documents.

The main responsibility of monitors is to help researchers ensure the ethics, scientificity and standardization of all aspects of the study. In accordance with GCP principles, monitors shall verify Case Report Forms (CRFs) against original data. Monitors will regularly contact each research center through on-site visits, letters or phone calls, and send representatives to assess research progress and subjects’ compliance with the trial protocol.

10. Data Storage

Completed CRFs and copies of examination forms will be submitted to the designated project team of Beijing Tiantan Hospital for verification, signature and filing. The project team shall properly store all received CRFs.

11. Data Safety Monitoring

All adverse events shall be fully recorded, properly handled and followed up until resolution or stable condition. Serious adverse events and unexpected events shall be promptly reported to the ethics committee and competent authorities in accordance with relevant regulations. The principal investigator shall conduct cumulative review of all adverse events regularly, and hold researcher meetings when necessary to evaluate the risk-benefit balance of the study and put forward suggestions on continuing the trial.

Subject data will be collected anonymously in CRFs during clinical research, and subjects can only be identified through subject numbers and pinyin name abbreviations. The signed informed consent form authorizes ethics committees and competent authorities to directly access the original data recorded in CRFs. All research personnel shall abide by professional confidentiality regulations and keep all personal identity and medical information of subjects strictly confidential.

12. Statistical Analysis

SPSS statistical software and R language will be used to establish databases and analyze clinical characteristic data; GraphPad Prism will be used for plotting. Measurement data with normal distribution will be compared between groups via t-test; non-normal measurement data will be analyzed via non-parametric rank-sum test. Categorical data will be compared via chi-square test or Fisher’s exact test. Mixed model analysis of variance will be used to compare intergroup differences at different time points. Partial correlation analysis will be conducted between brain network indicators and cognitive psychological indicators within each group, with comparative analysis against the HC group to evaluate the role of the cerebellum in language impairment and neuroplasticity mechanism. P < 0.05 is defined as statistically significant.

13. Ethical Considerations

This study can only be carried out after obtaining written approval from the ethics committee before subject recruitment. Before enrolling each subject, the principal investigator or designated researcher shall fully introduce the study purpose, procedures, potential risks and alternative treatment options to the subject or their legal representative, and obtain signed written informed consent. Subjects shall be clearly informed that they have the right to withdraw from the study at any time without penalty. All informed consent forms will be retained for inspection. Personal information of all subjects will be protected throughout the study.

14. Confidentiality of Data

The principal investigator holds all intellectual property rights of this project. All personal information of subjects will be strictly protected during data collection and analysis. Trial results will be published in accordance with institutional management regulations after database lock.
