# Supplementary material for: The application of cerebellar repetitive transcranial magnetic stimulation on aphasia after stroke: A randomized controlled trial protocol
Source: PLoS One. 2026 Aug 10;21(8):e0354837. doi: 10.1371/journal.pone.0354837 (PMC13456252; doi:10.1371/journal.pone.0354837)
Supplement: Supplementary file 3 [file pone.0354837.s001.doc]

SPIRIT 2025 Checklist: Recommended items to address in a clinical trial protocol and related documents*

| Section/Topic | No | Description |  | |
| --- | --- | --- | --- | --- |
| **Administrative information** | | |  | |
| Title and structured summary | 1a | Title stating the trial design, population, and interventions, with identification as a protocol (Page 1) |  | |
|  | 1b | Structured summary of trial design and methods, including items from the World Health Organization Trial Registration Data Set (Page 2-3) |  | |
| Protocol version | 2 | Version date and identifier (Page 1) |  | |
| Roles and responsibilities | 3a | Names, affiliations, and roles of protocol contributors (Page 1) |  | |
|  | 3b | Name and contact information for the trial sponsor (Page 1) |  | |
|  | 3c | Role of trial sponsor and funders in design, conduct, analysis, and reporting of trial; including any authority over these activities (Page 1) |  | |
|  | 3d | Composition, roles, and responsibilities of the coordinating site, steering committee, endpoint adjudication committee, data management team, and other individuals or groups overseeing the trial, if applicable (Page 1) |  | |
| **Open science** |  |  |  | |
| Trial registration | 4 | Name of trial registry, identifying number (with URL), and date of registration. If not yet registered, name of intended registry (Page 18) |  | |
| Protocol and statistical analysis plan | 5 | Where the trial protocol and statistical analysis plan can be accessed (Page 16) |  | |
| Data sharing | 6 | Where and how the individual de-identified participant data (including data dictionary), statistical code, and any other materials will be accessible (Page 17) |  | |
| Funding and conflicts of interest | 7a | Sources of funding and other support (e.g., supply of drugs) (Page 1) |  | |
|  | 7b | Financial and other conflicts of interest for principal investigators and steering committee members (Page 1) |  | |
| Dissemination policy | 8 | Plans to communicate trial results to participants, health care professionals, the public, and other relevant groups (e.g., reporting in trial registry, plain language summary, publication) (Page 17) |  | |
| Introduction |  |  |  | |
| Background and rationale | 9a | Scientific background and rationale, including summary of relevant studies (published and unpublished) examining benefits and harms for each intervention (Page 4-7) |  | |
|  | 9b | Explanation for choice of comparators (Page 4-7) |  | |
| Objectives | 10 | Specific objectives related to benefits and harms (Page 6-7) |  | |
| Methods: Patient and public involvement, trial design | | |  | |
| Patient and public involvement | 11 | Details of, or plans for, patient or public involvement in the design, conduct, and reporting of the trial (Page 8-9) |  | |
| Trial design | 12 | Description of trial design including type of trial (e.g., parallel group, crossover), allocation ratio, and framework (e.g., superiority, equivalence, non-inferiority, exploratory) (Page 8-9) |  | |
| **Methods: Participants, interventions, and outcomes** | | |  | |
| Trial setting | 13 | Settings (e.g., community, hospital) and locations (e.g., countries, sites) where the trial will be conducted (Page 9) |  | |
| Eligibility criteria | 14a | Eligibility criteria for participants (Page 9-10) |  | |
|  | 14b | If applicable, eligibility criteria for sites and for individuals who will deliver the interventions (e.g., surgeons, physiotherapists) (Page 10-11) |  | |
| Intervention and comparator | 15a | Intervention and comparator with sufficient details to allow replication including how, when, and by whom they will be administered. If relevant, where additional materials describing the intervention and comparator (e.g., intervention manual) can be accessed (Page 11) |  | |
|  | 15b | Criteria for discontinuing or modifying allocated intervention /comparator for atrial participant (e.g., drug dose change in response to harms, participant request, or improving /worsening disease) (Page 14) |  | |
| Outcomes | 16 | Primary and secondary outcomes, including the specific measurement variable (e.g., systolic blood pressure), analysis metric (e.g., change from baseline, final value, time to event), method of aggregation (e.g., median, proportion), and time point for each outcome (Page 11-13) |  | |
| Harms | 17 | How harms are defined and will be assessed (e.g., systematically, non-systematically) (Page 14) |  | |
| Participant timeline | 18 | Time schedule of enrolment, interventions (including any run-ins and washouts), assessments, and visits for participants. A schematic diagram is highly recommended (see Figure) (Fig 1, Table 1) |  | |
| Sample size | 19 | How sample size was determined, including all assumptions supporting the sample size calculation (Page 10) |  | |
| Recruitment | 20 | Strategies for achieving adequate participant enrolment to reach target sample size (Page 10) |  | |
| **Methods: Assignment of interventions** | | |  | Strategies to improve adherence to intervention protocols, and any procedures for monitoring adherence (eg, drug tablet return, laboratory tests) (Page 6) |
| Randomization: |  |  |  | |
| Sequence generation | 21a | Who will generate the random allocation sequence and the method used (Page 10-11) |  | |
|  | 21b | Type of randomization (simple or restricted) and details of any factors for stratification. To reduce predictability of a random sequence, other details of any planned restriction (e.g., blocking) should be provided in a separate document that is unavailable to those who enrol participants or assign Interventions (Page 10-11) |  | |
| Allocation concealment mechanism | 22 | Mechanism used to implement the random allocation sequence (e.g., central computer / telephone; sequentially numbered, opaque, sealed containers), describing any steps to conceal the sequence until interventions are assigned (Page 10-11) |  | |
| Implementation | 23 | Whether the personnel who will enrol and those who will assign participants to the interventions will have access to the random allocation Sequence (Page 10-11) |  | |
| Blinding | 24a | Who will be blinded after assignment to interventions (e.g., participants, care providers, outcome assessors, data analysts) (Page 10-11) |  | |
|  | 24b | If blinded, how blinding will be achieved and description of the similarity of interventions (Page 10-11) |  | |
|  | 24c | If blinded, circumstances under which unblinding is permissible, and procedure for revealing a participant’s allocated intervention during the trial (Page 10-11) |  | |
| **Methods: Data collection, management, and analysis** | | |  | |
| Data collection methods | 25a | Plans for assessment and collection of trial data, including any related processes to promote data quality (e.g., duplicate measurements, training of assessors) and a description of trial instruments (e.g., questionnaires, laboratory tests) along with their reliability and validity, if known. Reference to where data collection forms can be accessed, if not in the protocol (Page 15-16) |  | |
|  | 25b | Plans to promote participant retention and complete follow- up, including list of any outcome data to be collected for participants who discontinue or deviate from intervention protocols (Page 15-16) |  | |
| Data management | 26 | Plans for data entry, coding, security, and storage, including any related processes to promote data quality (e.g., double data entry; range checks for data values). Reference to where details of data management procedures can be accessed, if not in the Protocol (Page 16) |  | |
| Statistical methods | 27a | Statistical methods used to compare groups for primary and secondary outcomes, including harms (Page 14-15) |  | |
|  | 27b | Definition of who will be included in each analysis (e.g., all randomized participants), and in which group (Page 14-15) |  | |
|  | 27c | How missing data will be handled in the analysis (Page 14-15) |  | |
|  | 27d | Methods for any additional analyses (e.g., subgroup and sensitivity analyses) (Page 14-15) |  | |
| Methods: Monitoring | | |  |  |
| Data monitoring committee | 28a | Composition of data monitoring committee (DMC); summary of its role and reporting structure; statement of whether it is independent from the sponsor and funder; conflicts of interest and reference to where further details about its charter can be found, if not in the protocol. Alternatively, an explanation of why a DMC is not needed (Page 16) |  | |
|  | 28b | Explanation of any interim analyses and stopping guidelines, including who will have access to these interim results and make the final decision to terminate the trial (Page 16) |  | |
| Trial monitoring | 29 | Frequency and procedures for monitoring trial conduct. If there is no monitoring, give explanation (Page 16-17) |  | |
| **Ethics** |  |  |  | |
| Research ethics approval | 30 | Plans for seeking research ethics committee / institutional review board approval (Page 17-18) |  | |
| Protocol amendments | 31 | Plans for communicating important protocol modifications to relevant parties (Page 17-18) |  | |
| Consent or assent | 32a | Who will obtain informed consent or assent from potential trial participants or authorized proxies, and how (Page 17-18) |  | |
|  | 32b | Additional consent provisions for collection and use of participant data and biological specimens in ancillary studies, if applicable (Page 17-18) |  | |
| Confidentiality | 33 | How personal information about potential and enrolled participants will be collected, shared, and maintained in order to protect confidentiality before, during, and after the trial (Page 17-18) |  | |
|  |  |  |  | |

*It is strongly recommended that this checklist be read in conjunction with the SPIRIT 2025 Explanation & Elaboration for important clarification on the items. Amendments to the protocol should be tracked and dated. The SPIRIT checklist is copyrighted by the SPIRIT Group under the Creative Commons “[Attribution-NonCommercial-NoDerivs 3.0 Unported](http://www.creativecommons.org/licenses/by-nc-nd/3.0/)” license.

1. Chan AW, Boutron I, Hopewell S, et al. SPIRIT 2025 Statement: Updated Guideline for Protocols of Randomized Trials. JAMA. 2025;334(5):435-443. doi:10.1001/jama.2025.4486
