# Supplementary material for: The application of cerebellar repetitive transcranial magnetic stimulation on aphasia after stroke: A randomized controlled trial protocol
Source: PLoS One. 2026 Aug 10;21(8):e0354837. doi: 10.1371/journal.pone.0354837 (PMC13456252; doi:10.1371/journal.pone.0354837)
Supplement: Supplementary file 4 [file pone.0354837.s003.doc]

项目来源与编号：国家自然科学基金 82572919

**以小脑为靶点的经颅磁刺激技术在卒中后运动性失语患者语言康复中的作用及脑网络机制研究方案**

所属项目名称：以小脑为靶点的经颅磁刺激技术在卒中后运动性失语患者语言康复中的作用及脑网络机制研究

项目负责人/承担科室：张玉梅/康复医学科

课题委托单位：首都医科大学附属北京天坛医院

课题承担单位：首都医科大学附属北京天坛医院

课题主持单位：首都医科大学附属北京天坛医院

研究年限：2026年1月 － 2029年12月

版本号：V1.0

版本日期：2026年1月8日

方 案 摘 要

| **项目名称** | 以小脑为靶点的经颅磁刺激技术在卒中后运动性失语患者语言康复中的作用及  脑网络机制研究 |
| --- | --- |
| **研究目的** | 1.阐明小脑在卒中后运动性失语患者语言功能损害中的作用。  2.明确以右侧小脑为靶点的经颅磁刺激治疗对卒中后失语患者的语言康复效果。  3.探讨小脑通过大脑-小脑环路调控语言功能的动态脑网络可塑性机制。 |
| **研究设计** | 前瞻性、随机对照研究 |
| **病例总数** | 1.本研究第一部分为观察组，拟入组观察组卒中后运动性失语患者共 44 例，健康对照组 44 例。  2.本研究第二部分为干预组，拟入组卒中后运动性失语患者共60例。  综上，本项目拟入组病例数110例，健康对照44例。 |
| 病例选择 | *入选标准：*   1. 年龄18岁至80周岁(包含18岁和80岁)，小学及以上文化程度； 2. 母语为标准普通话，右利手，无左利手家族史： 3. 病程介于14至60天，发病时以语言障碍为主； 4. 第二部分研究排除存在TMS禁忌症的患者； 5. 首次发病，经CT/MRI证实为脑卒中，病变位于左侧大脑半球且病灶单一； 6. 经西部失语成套测验评定为BA:采用洛文斯顿认知功能评定测试评定患者的认知功能，排除失语症合并严重非语言认知功能损害的患者； 7. 能够配合完成神经心理学测评以及影像学检查； 8. 签署知情同意书。 |
|  | *排除标准*  1. 本次发病前已有语言障碍，或合并其它原因导致的语言障碍：短暂性脑缺血发作病史：烟雾病，淀粉样血管变性等与认知功能障碍相关的病因引起的脑卒中；2. 有精神分裂症等精神疾病病史，第二部分研究排除存在TMS禁忌症的患者；3. 合并严重的心，肝，肾功能不全：合并视野缺损或外周听力障碍：  4. 有开颅手术使或既往有癫痫发作；  5. 合并严重构音障碍：合并意识障碍格拉斯哥昏迷评分15分；  6. 影像学发现脑白质病变，微出血，血管周围间隙扩大，脑萎缩等脑小血管病变；  7. 或本次发病后合并抑郁状态(医院版卒中后失语抑郁问卷(第10版)评分〉6分)；  8. 研究过程中，卒中再次复发或颅内产生新的病灶(占位等)；  9. 签署知情同意书；  10. 无法完成TMS治疗；  11. 磁共振扫描过程中，头部移动明显(>3mm)，影响图像效果；  12. 不能完成随访；  13. 患者主动要求退出研究。 |
| **治疗方案** | （一）基线信息登记（入组时）  ①人口统计学信息：年龄、性别、利手、文化程度、居住情况；  ②卒中危险因素：高血压、糖尿病、高脂血症、冠心病、吸烟、体重指数、血常规、肝功能、肾功能、同型半胱氨酸水平；  ③脑血管病相关信息：卒中发病日期、卒中类型、病灶部位；  （二）语言、认知、脑影像评估（入组 7 天内）  1.量表评估  ①爱丁堡利手量表  ②语言功能评估：西方失语症成套测验量表、语言流畅性测试、音位水平测试、词汇水平测试、语法水平测试（句子产生）、波士顿失语诊断测验  ③工具性日常生活活动能力量表、卒中和失语症生活质量量表  2.脑影像评估  头部 MRI（T1WI、T2WI、T2-FLAIR、SWI、DTI、rs-fMRI）  （三）康复治疗  受试者入组后按照随机原则被分配至以下两组：  ①常规语言康复训练+以右侧小脑为刺激靶点真刺激组；  ②常规语言康复训练+以右侧小脑为刺激靶点假刺激组；  **康复方案**：每日上、下午各进行 1 次语言康复训练，其中常规语言康复训练 30  分钟，经颅磁刺激采用 cTBS 模式，刺激约 1 分钟（40 秒），总计 31 分钟，每周进行 5 天语言康复训练，共 4 周。  **（1）常规语言康复训练：**由北京天坛医院语言康复治疗师对患者进行一对一结合计算机辅助的语言功能训练，具体任务主要针对言语流畅性、音位水平、词汇 水平、语法水平、听理解及口语表达等方面进行个体化训练。同时，应用我们前期已经完成的国家自然科学基金的研究成果（81972144），利用患者残存的语言功能进行训练。  **（2）TMS 治疗方案：**采用磁刺激仪（型号：YRD CCY-Ⅰ）对 BA 患者进行  cTBS 治疗，刺激治疗时采用 8 字型线圈。刺激参数：丛内频率为 50Hz，丛间频率 为 5Hz，强度为 80%健侧大脑半球运动阈值（运动阈值为刺激运动皮层手区 10 次，能够观察到至少 5 次手部抽动的最小磁场强度），时间为40s，脉冲总数为 600 个。假刺激组采用同样参数的伪刺激脉冲。  体表定位采用国际脑电图学会标定的电极定位：  ①真刺激组的靶点为右侧小脑，体表定位点为枕骨粗隆下 1cm、右旁开 3cm 处（The Joumal Physiology,2015,593(22):4963-4977.），线圈位置与头皮相切。  ②假刺激组的靶点同真刺激组，线圈位置与头皮相切。 |
| **疗效评定** | 有效性评价指标（主要疗效指标和次要疗效指标）  1.主要评价指标  ①小脑在卒中后 BA 患者语言功能损害中的作用。  ②小脑通过大脑-小脑环路调控语言功能的动态脑网络可塑性机制。  2.次要评价指标  ①评估不同康复训练方案的有效性。  ②了解随访过程中影响语言功能恢复的因素包括教育程度、卒中严重程度等因素。 |
|  | *安全性评价指标*  在每次经颅磁刺激治疗过程中及治疗结束后，对所有患者以问卷调查的形式评估 TMS 治疗是否引起了局部灼烧感、头部不适或疼痛、面部下颌肌肉抽搐、听觉敏感、耳鸣、晕厥、情绪波动、注意力不集中、癫痫发作等不良反应。如果患者出现安全性问题，研究人员会及时进行对症处理。 |
| **统计方法** | 1. 基线指标的组间比较   采用SPSS 25.0统计学软件建立数据库并进行统计学分析。对数据进行正态性检验，计量资料（如各向异性值、节点度值等）使用“均数 ±标准差”或“中位数（下四分位数，上四分位数）”表示，组间比较使用 t 检验或Wilcoxon、K-W 秩和检验；计数资料使用“率”表示，组间比较使用卡方检验、Fisher's精确概率法或 Wilcoxon 秩和检验。P＜0.05 认为统计结果具有显著性差异。   2. 有效性分析   ①临床指标：根据意向性分析（Intention-To-Treat analysis，ITT）的基本原则，采用重复测量方差分析或 Wilcoxon 秩和检验评价各组别康复治疗前后不同时间点受试者语言功能的改善情况，评价以右侧小脑为靶点 TMS 组和以右侧小脑为靶点假刺激组两组间康复效果的差异。P＜0.05 认为统计结果具有显著性差异。  ②影像学指标：将 BA 组（包括不同组别治疗前后）的语言功能评分分别与 FA 值、节点度值、ALFF 值、功能连接密度（Functional Connectivity Density，FCD）  值等进行 Pearson 相关分析、动态功能连接密度分析及聚类分析，并与 HC 组进行 对比分析，以评价小脑在语言功能损伤及神经可塑性机制中的作用。经 FDR 校正，q＜0.05 的体素为显著。 |
| **研究期限** | 2026.1-2029.12 |

**一、研究背景**

脑卒中是危害我国国民健康的重大疾病之一，来自北京天坛医院的全国多中心大样本研究，验证了脑卒中具有高发病率、高致残率、高死亡率及高复发率的特点。卒中后多数患者遗留功能障碍，其中失语症是常见的功能障碍之一，发生率约为 21-42%。如何改善失语症患者的语言功能，提高患者的沟通能力，是目前脑卒中研究领域迫切需要解决的问题之一。 既往研究已发现，应用经颅磁刺激（Transcranial Magnetic Stimulation, TMS） 治疗可促进失语症患者语言功能的恢复。基于 Meta 分析及国内外专家共识或指南，针对卒中后失语（post-stroke aphasia, PSA）患者，TMS 靶点主要推荐病灶 周围部位和病灶对侧周围镜像区，但这两个靶点均存在一定的局限性。目前，关于小脑对语言功能的调控作用受到了广泛的关注。研究发现小脑与额 叶皮层之间存在广泛的神经环路，小脑功能具有偏侧化：左侧小脑通过更多地连接前额叶、顶叶、皮层下大脑区域在认知控制中发挥重要作用，而右侧小脑则更多地参与语言控制。因此，小脑具有独特的可塑性机制和与皮质区域的广泛连接，有望成为失语症康复的潜在目标。本课题组的前期研究发现：失语症患者存在广泛纤维联系的改变，其中包含与小脑纤维联系的变化。因此，课题组推测：小脑可通过大脑-小脑环路在 PSA 患者的语言功能损伤中发挥作用，尤其是右侧小脑，有望成为TMS 治疗失语症的潜在靶点。然而，目前关于小脑 TMS 治疗 PSA 的研究或因样本量较少，小脑 TMS 干预 后症状改善不显著；或因随访条件受限，没有对治疗后的患者进行不同时期疗效的观察，导致以小脑为靶点的 TMS 治疗对 PSA 患者语言功能的康复效果及其脑网络可塑性机制尚无统一定论。

因此，目前急需开展相关的基础及临床研究，探讨小脑通过大脑-小脑环路在PSA患者语言功能中的脑网络机制；开展随机对照研究，动态观察以右侧小脑为 靶点的TMS治疗后，不同时间点PSA患者语言功能的恢复情况及脑网络的可塑 性变化，进一步探讨小脑与语言网络之间的联系，挖掘小脑作为TMS刺激靶点的潜力，改善失语患者的语言功能预后。

**二、研究目的**

**1. 主要目的：**

（1）阐明小脑在卒中后BA患者语言功能损害中的作用。

（2）明确以右侧小脑为靶点的TMS治疗对BA患者的语言康复效果。

（3）探讨小脑通过大脑-小脑环路调控语言功能的动态脑网络可塑性机制。

**2. 次要目的：**

（1）阐明小脑在卒中后BA患者语言功能损害中的作用，重点观察小脑额叶功能连接强度变化

（2）探索以右侧小脑为靶点的TMS治疗对BA患者语言康复的效果及脑网络可塑性机制

**三、研究设计类型、原则与试验步骤**

**1. 研究设计**

本研究分两部分完成。第一部分拟入组卒中后BA患者作为观察组，为阐明小脑参与卒中后运动性失语（Broca's aphasia，BA）患者语言功能损害的脑网络机制。第二部分拟入组卒中后BA患者，接受康复训练为明确以小脑为靶点行TMS治疗对BA患者语言功能的康复疗效及脑网络可塑性机制，，均以右侧小脑为TMS靶点，采用计算机随机化方法分为真刺激组和假刺激组。

**2. 样本量及研究计划**

第一部分为阐明小脑参与卒中后运动性失语（Broca's aphasia，BA）患者语言功能损害的脑网络机制，拟入组卒中后BA患者作为观察组，筛选年龄、性

别、利手、文化程度相匹配的健康人群作为HC组。

其中α=0.05，Z（（1-α）/2）=1.96，容许误差δ=0.1，p代表所设计的语言任务适用于BA患者的特异度。综合既往语言学研究招募的受试人群数目，拟入组BA患者共40人，考虑患者失访及脱落等情况，增加10%样本量，故本研究第一部分拟入组观察组BA患者共44例，HC组44例。

第二部分为明确以小脑为靶点行TMS治疗对BA患者语言功能的康复疗效及脑网络可塑性机制，拟入组卒中后BA患者，均以右侧小脑为TMS靶点，采用计算机随机化方法分为真刺激组和假刺激组。

采用成组设计、两组病例数相等、双侧差异性检验：

其中，α=0.05，β=0.20，p1、p2分别代表以右侧小脑为靶点TMS组、假刺激组在刺激后3个月语言功能的改善率。综合既往语言学研究招募的受试人群数目，拟入组BA患者共60人（以右侧小脑为靶点 TMS 组和假刺激组分别 30 人），考虑患者失访及脱落等情况，增加10%样本量，故本研究第二部分拟入组BA患者共66例。

综上，本研究所需总样本量为：BA组（44+66）110例，HC组44例。

3. **研究期限**

本研究为 2026 年 1 月至 2029 年 12 月。

4. **受试者选择**

BA患者入组标准：①年龄18岁至80周岁(包含18岁和80岁)，小学及以上文化程度；②母语为标准普通话，右利手，无左利手家族史；③病程介于14至60天，发病时以语言障碍为主；④第二部分研究排除存在TMS禁忌症的患者；⑤首次发病，经CT/MRI证实为脑卒中，病变位于左侧大脑半球且病灶单一；⑥经西部失语成套测验评定为BA:采用洛文斯顿认知功能评定测试评定患者的认知功能，排除失语症合并严重非语言认知功能损害的患者；⑦能够配合完成神经心理学测评以及影像学检查；⑧签署知情同意书；

BA患者排除标准：①本次发病前已有语言障碍，或合并其它原因导致的语言障碍：短暂性脑缺血发作病史：烟雾病，淀粉样血管变性等与认知功能障碍相关的病国引起的脑卒中；②有精神分裂症等精神疾病病史，第二部分研究排除存在TMS禁忌症的患者；③合并严重的心，肝，肾功能不全：④合并视野缺损或外周听力障碍：⑤有开颅手术使或既往有癫痫发作；合并严重构音障碍：合并意识障碍格拉斯哥昏述评分15分；⑥影像学发现脑白质病变，微出血，血管周围间隙扩大，脑萎缩等脑小血管病变；⑦或本次发病后合并抑郁状态(医院版卒中后失语抑郁问卷(第10版)评分〉6分)；⑧研究过程中，卒中再次复发或颅内产生新的病灶(占位等)；⑨签署知情同意书；⑩无法完成TMS治疗；磁共振扫描过程中，头部移动明显(>3mm)，影响图像效；不能完成随访；患者主动要求退出研究；

HC入组标准：①小学及以上文化程度；②无脑血管疾病、脑外伤、癫痫、痴呆或其他神经系统疾病病史；③心、肺、肝、肾等脏器功能正常；④无视野缺损或外周听力障碍；⑤经西部失语成套测验评定语言功能正常（失语商＞93.8分）且经蒙特利尔认知评估量表评估认知功能正常（总分≥26分）；⑥无MRI检查禁忌症；MRI检查未见异常；⑦签署知情同意书；

HC排除标准：①有脑卒中、颅内占位等疾病史;②有精神分裂症等明确精神疾病病史，或合并抑郁状态；

1. **研究方法**

第一部分为观察性研究，拟通过对卒中后BA患者进行行为学及影像学评估，探讨小脑在BA患者语言功能损伤中发挥的作用及脑网络机制，验证右侧小脑调控语言功能的偏侧化，为选择以右侧小脑为TMS刺激靶点奠定理论基础（如下图所示）。

第二部分为随机对照临床研究。拟通过对卒中后BA患者以右侧小脑为靶点进行TMS治疗，动态观察小脑参与语言功能恢复的脑网络可塑性机制（如下图所示）。

1.神经心理学评估

在第一部分中，对BA患者和HC组在治疗前即基线±7天进行神经心理学评估；

在第二部分中，对BA患者在卒中后的不同阶段（治疗前即基线±7天，治疗后2周±3天，治疗后1个月±7天，治疗后3个月±14天，共4个时间点）进行神经心理学评估。在卒中后的不同阶段，设计相应的语言任务，依据语言功能的演变特点，具体语言任务有差异，进一步明确BA患者在卒中后的不同阶段语言功能损害的特点，并排除患者由于对语言任务的记忆功能对结果产生的影响。

（1）口语产出功能评估：

①口语产出流畅性任务

语言流畅性测试（Verbal Fluency Test, VFT）：要求被试在一定时间内以一定规则自发说出一定数量的词语并避免重复（如以某个声母开头的词或说出几种动物的名称等），考察被试的语义流畅性、语音流畅性和动词流畅性。

西方失语症成套测验（Western Aphasia Battery, WAB）：采用WAB量表的评价结果，使用该量表中的子测验自发性言语部分对BA患者进行评估，并计算流畅度得分。

②不同水平的口语产出任务

音位水平测试：使用华东师范大学的汉语构音能力测验“黄昭鸣-韩知娟词表”（梁峻波,黄昭鸣.学龄痉挛型脑瘫儿童语音清晰度特征及其与言语子系统的相关性研究.[硕士]2016.）。测试材料由50个单音节词组成，包含21个声母、13个韵母和4个声调。通过音位对比、最小音位对比和音位习得情况的分析，评估被试在音位水平上口语产出功能。

词汇水平测试：采用北京师范大学语料库中的词汇水平测验。该测验向被试呈现一系列黑白图片并要求其进行命名，测验包含物体命名和动作命名。测试材料为快速自动化命名测验（Dyslexia (Chichester, England).2018,24(3):276-93.）此外，采用波士顿命名测试考察BA患者的命名能力。

语法水平测试（句子产生）：该测验包括句子产生和句子理解两部分。句子产出部分使用北京语言大学与美国西北语言大学联合开发的动词和语句量表（中国听力语言康复科学杂志,2017,15(05):336-41.）中的句子产生启动子测验来进行评估，主要评估动词和动词论元结构产生的障碍。句子理解部分使用中国康复研究中心汉语标准失语症检查表中的“句子-图片匹配”子测验。

（2）听理解及口语表达等语言功能评估：

采用WAB量表的评价结果，其中量表中子测验听理解部分主要评估被试的口语理解能力；子测验复述部分主要评估被试的复述能力；子测验阅读部分和书写部分主要评估被试的阅读、书写能力。

（3）失语严重程度评估：

采用波士顿失语诊断测验（Boston Diagnostic Aphasia Examination, BDAE）对BA患者失语的严重程度进行分级。

（4）工具性日常生活活动能力和生活质量评估：

采用工具性日常生活活动能力量表（Instrumental Activities of Daily Living Scale, IADL）及卒中和失语症生活质量量表（Stroke and Aphasia Quality of Life Scale, SAQOL-39g）考察BA患者的工具性日常生活活动能力和生活质量。

2.影像学数据采集

利用GE Signa HDxt 3.0T磁共振及8通道头部线圈，对BA组患者分别在3个时间点（基线±7天，治疗后2周±3天、1个月±7天）进行影像学检查，并采集HC组数据1次。影像学数据采集均由一台磁共振机器扫描完成。扫描过程中用橡胶耳塞降低噪音，用泡沫垫固定头位减少运动伪影。在静息态功能磁共振成像（resting state functional MRI，rs-fMRI）扫描过程中，要求被试闭上双眼保持清醒。

影像学数据采集序列包括：T1WI、T2WI、T2-FLAIR、SWI、DTI、rs-fMRI。

具体参数设定如下：

| 表1 MRI各序列参数设定 | | | | | | | | |
| --- | --- | --- | --- | --- | --- | --- | --- | --- |
| 扫描序列 | TR  ms | TE  ms | FOV  mm^2^ | 层厚 mm | 层间距 mm | 层数 | 矩阵大小 | 其他 |
| T1WI | 2500 | 4.37 | 256x256 | 0.8 | 0 | 208 | 256x256 | — |
| T2WI | 4500 | 84 | 220x220 | 1 | 0.5 | 28 | 256x256 | — |
| T2-FLAIR | 7800 | 91 | 199x220 | 5 | 5 | 24 | 256x256 | — |
| SWI | 27 | 20 | 230x230 | 2.6 | 0 | 48 | 256x256 | — |
| DTI | 13000 | 69.3 | 250x250 | 3.5 | 0 | 36 | 128x128 | 方向数30 b=0和1500s/mm |
| rs-fMRI | 650 | 27 | 200x200 | 2 | 0.7 | 72 | 100x100 | — |

3.康复训练

将第二部分中入组的BA患者随机分为两组进行康复训练：

①常规语言康复训练+以右侧小脑为刺激靶点真刺激组；

②常规语言康复训练+以右侧小脑为刺激靶点假刺激组；

康复方案：每日上、下午各进行1次语言康复训练，其中常规语言康复训练30分钟，经颅磁刺激采用cTBS模式，刺激约1分钟（40秒），总计31分钟，每周进行5天语言康复训练，共4周。

（1）常规语言康复训练：由北京天坛医院语言康复治疗师对患者进行一对一结合计算机辅助的语言功能训练，具体任务主要针对言语流畅性、音位水平、词汇水平、语法水平、听理解及口语表达等方面进行个体化训练。同时，应用我们前期已经完成的国家自然科学基金的研究成果（81972144），利用患者残存的语言功能进行训练。

（2）TMS治疗方案：采用磁刺激仪（型号：YRD CCY-Ⅰ）对BA患者进行cTBS治疗，刺激治疗时采用8字型线圈。刺激参数：丛内频率为50Hz，丛间频率为5Hz，强度为80%健侧大脑半球运动阈值（运动阈值为刺激运动皮层手区10次，能够观察到至少5次手部抽动的最小磁场强度），时间为40s，脉冲总数为600个。假刺激组采用同样参数的伪刺激脉冲。

体表定位采用国际脑电图学会标定的电极定位：

①真刺激组的靶点为右侧小脑，体表定位点为枕骨粗隆下1cm、右旁开3cm处（The Joumal Physiology,2015,593(22):4963-4977.），线圈位置与头皮相切。

②假刺激组的靶点同真刺激组，线圈位置与头皮相切。

**五、伴随用药**

本研究不涉及临床药物使用，不限制患者口服药物治疗。

**六、观察指标与检查时间**

（1）符合入组标准的受试者在卒中后 2 周内接受语言及生活能力功能的评估，包括：①西方失语症成套测验；②音位水平测试；③爱丁堡利手调查问卷；③词汇水平测试；④语法水平测试；⑤言语流畅性试验；⑥日常生活活动能力量表；⑦ 日常生活活动能力量表⑧工具性日常生活活动能力量表；

（2）入组患者在卒中后 2 周内采用北京天坛医院西门子 3.0T 磁共振采集 影像学数据，主要扫描序列包括：T1WI、T2WI、3D-FLAIR、rs-fMRI、DTI。

（3） 随访：入组患者在康复训练结束后 3 天内、康复训练 1 个月±7 天、康复训练 3 个月±14 天进行语言功能等行为学数据的采集。在康复训练结束后 3 天内、康复训练 1 个月±7 天进行影像学数据的采集。

**七、随访时间**

在治疗前即基线±7天、治疗后2周±3天、1个月±7天、3个月±14天进行语言功能等行为学数据的采集，在基线±7天、治疗后2周±3天、1个月±7天进行影像学数据的采集。

1. **临床评价**
1. 客观疗效评价：根据意向性分析（Intention-To-Treat analysis，ITT）的基本原则，采用重复测量方差分析或Wilcoxon秩和检验评价各组别康复治疗前后不同时间点受试者语言功能的改善情况，评价以右侧小脑为靶点TMS组和以右侧小脑为靶点假刺激组两组间康复效果的差异。P＜0.05认为统计结果具有显著性差异。
2. 安全性评价：在每次经颅磁刺激治疗过程中及治疗结束后，对所有患者以问卷调查的形式评估TMS治疗是否引起了局部灼烧感、头部不适或疼痛、面部下颌肌肉抽搐、听觉敏感、耳鸣、晕厥、情绪波动、注意力不集中、癫痫发作等不良反应。如果患者出现安全性问题，研究人员会及时进行对症处理。
4. **项目已知和潜在的风险和获益，以及对风险的处置预案**

本研究涉及经颅磁刺激治疗和语言/认知康复训练。经颅磁刺激可能引起 的不良反应如下：局部烧灼感、头痛、耳鸣、焦虑、抑郁、癫痫等。语言/认知康复训练无相关不良反应。此外可能涉及一些安全事件，如跌倒、卒中再发等。上述在经颅磁刺激治疗中出现的所有症状均为本研究的不良事件，癫痫发作为严重不良事件。出现不良事件后应及时告知家属及患者，完善头部影像学检查判断有无原发疾病进展可能，详细记录不良事件情况，3 日内召开研究内部小组讨论会，决定是否为本次研究终点。出现严重不良事件后应及时采取医疗手段治疗，完善头部影像学检查，告知家属，记录严重不良事件情况，1 日内召开研究内部小组讨论会，研究中止。

1. **研究的质量控制与质量保证**

研究者保证在进行临床研究时遵循临床试验方案，遵循临床研究管理规范以及相应法律法规。研究者应保证遵循临床研究方案中的所有研究操作。研究者应提供可靠数据和临床试验方案要求的所有信息，并确保监查团队可直接查看原始资料。监查团队的主要责任是帮助研究者保证临床研究各个方面的伦理性、科学性、专业性和规范性。根据 GCP 原则，监督团队应对照原始资料对CRF 进行核查。监查团队将定期通过现场访视、信件或电话形式与每个中心联系，将派出代表来评估研究进展、研究者和受试者对临床方案的依从性。

1. **资料保存**

病例报告表、检查单复印件由研究者递送至首都医科大学附属北京天坛医院指定的项目组，项目组相关负责人员进行核对签收，项目组应对收到的病例报告表进行妥善保存。

**十二、数据安全监查**

所有不良事件均详细记录，恰当处理并追踪直到妥善解决或病情稳定，按照规定及时向伦理委员会、主管部门和监督管理部门报告严重不良事件与非预期事件等；主要研究者定期对所有不良事件进行累积性回顾，必要时召开研究者会议评估研究的风险与受益，做出研究是否继续进行的建议。临床研究过程中，受试者的数据应以匿名方式收集在病例报告表上，受试者只有通过受试者编号和姓名拼音缩写来辨认。知情同意书允许受试者同意已授权的伦理委员会、权威机构直接查阅病例报告表上相关的原始资料。所有参与人员应遵循职业保密规定，必须对受试者的所有个人身份信息或医疗信息保密。

**十三、统计学处理**

采用 SPSS 统计学软件和 R 语言建立数据库，进行临床特征数据分析，采 用 GraphPad Prism 软件进行作图。计量资料如为正态分布，组间比较采用 t 检 验，不符合正态分布者，组间比较采用非参数秩和检验。分类资料组间比较采 用卡方检验或 Fisher’s 确切概率法。对于不同组被试的组间差异比较采用混 合模型方差分析。同时，在各组组内将脑网络属性指标和认知心理指标进行偏相关分析，并与HC组进行对比分析，以评价小脑在语言功能损伤及神经可塑性机制中的作用。p<0.05 认为统计结果具有显著性差异。

**十四、伦理考虑**

在研究开始之前，由伦理委员会批准该试验方案后才实施临床研究。每一位受试者入选本研究前，研究者有责任向受试者或其合法代理人全面地介绍本研究的目的、程序和可能的风险，以及替代治疗的相应信息，并签署书面知情同意书，应让受试者知道他们有权随时退出本研究，知情同意书作为临床研究文件保留备查。研究过程中将保护受试者的个人信息不被泄露。

**十五、资料保密**

主要研究者拥有完整的知识产权。整个研究过程及数据分析过程均严格保护受试者信息，执行委员会将按照管理条例和程序来公布该试验的结果。数据库锁定后试验结果将尽快被公布。

十六、参加人员

| **姓名** | **职称/专业** | **任务** | **GCP培训**  **（时间）** |
| --- | --- | --- | --- |
| 张玉梅 | 主任医师 | 课题统筹与协调 | 2019.12 |
| 逄文斌 | 讲师 | 影像学数据分析 | 无 |
| 刘朝曦 | 医师 | 影像学数据收集 | 无 |
| 尹志科 | 副主任护师 | 康复护理 | 2025.06 |
| 向春晨 | 医师 | 筛选患者，磁刺激操作 | 2025.06 |
| 吴娱倩 | 技师 | 磁刺激操作 | 无 |
| 李越秀 | 医师 | 筛选患者，统计学分析 | 2025.06 |
| 李丽 | 主管技师 | 量表评定及康复训练 | 2023.12 |
| 赵然 | 主管护师 | 康复护理 | 无 |

1. **参考文献**

[1] Xiong Y, Campbell BCV, Schwamm LH, Meng X, Jin A, Parsons MW, Wang Y. Tenecteplase for Ischemic Stroke at 4.5 to 24 Hours without Thrombectomy. The New England journal of medicine. 2024;391(3):203-12

[2] The Rehabilitation and recovery of people with Aphasia after Stroke (RELEASE) Collaborators. Dosage, Intensity, and Frequency of Language Therapy for Aphasia: A Systematic Review-Based, Individual Participant Data Network Meta-Analysis [J]. Stroke, 2022, 53(3): 956-67.

[3] Ding X, Zhang S, Huang W, et al. Comparative efficacy of non-invasive brain stimulation for post-stroke aphasia: A network meta-analysis and meta-regression of moderators [J]. Neuroscience and biobehavioral reviews, 2022, 140: 104804.

***[4] 中华医学会神经病学分会神经康复学组, 中国康复医学会脑血管病专业委员会, 中国康复研究中心. 卒中后失语临床管理专家共识[J]. 中国康复理论与实践, 2022, 28(1): 15-23.***

[5] Gholami M, Pourbaghi N, Taghvatalab S. Evaluation of rTMS in patients with poststroke aphasia: a systematic review and focused meta-analysis. Neurological sciences: official journal of the Italian Neurological Society and of the Italian Society of Clinical Neurophysiology. 2022;43(8):4685-94.

[6] Wang C, Nie P, Wang P, Wang Y, Zang Y, Zhang Y. The Therapeutic Effect of Transcranial Magnetic Stimulation on Post-stroke Aphasia and the Optimal Treatment Parameters: A Meta-analysis. Archives of physical medicine and rehabilitation. 2024;105(7):1388-98.

[7] Harvey D, Hamilton R. Noninvasive brain stimulation to augment language therapy for poststroke aphasia [J]. Handbook of clinical neurology, 2022, 185: 241-50.

[8] Anglade C, Thiel A, Ansaldo AI. The complementary role of the cerebral hemispheres in recovery from aphasia after stroke: a critical review of literature [J]. Brain Inj, 2014, 28: 138-145.

[9] Chen Z, Zhang R, Huo H, et al. Functional connectome of human cerebellum [J]. NeuroImage, 2022, 251: 119015.

[10] Arleo A, Bares M, Bernard J A, et al. Consensus Paper: Cerebellum and Ageing [J]. Cerebellum (London, England), 2024, 23(2): 802-32.

[11] Yuan Q, Li H, Du B, et al. The cerebellum and cognition: further evidence for its role in language control [J]. Cerebral cortex (New York, NY: 1991), 2022, 33(1): 35-49.

[12] van Dun K, Bodranghien F, Manto M, et al. Targeting the Cerebellum by Noninvasive Neurostimulation: a Review [J]. Cerebellum, 2017, 16: 695-741.

[13] van Dun K, Mitoma H, Manto M. Cerebellar Cortex as a Therapeutic Target for Neurostimulation [J]. Cerebellum, 2018, 17: 777-787.

***[14] Liu Q, Zhang Y. A Comparative Study on Cognitive Assessment in Cerebellar and Supratentorial Stroke. Brain sciences. 2024;14(7).***

[15] International Brain Initiative: An Innovative Framework for Coordinated Global Brain Research Efforts. Neuron. 2020;105(2):212-6.

[16] Tilton-Bolowsky V, Stockbridge M D, Hilis A E. Remapping and Reconnecting the Language Network after Stroke [J]. Brain sciences, 2024, 14(5).

[17] Zhang X, Chen Z, Li N, et al. Regional Alteration within the Cerebellum and the Reorganization of the Cerebrocerebellar System following Poststroke Aphasia [J]. Neural plasticity, 2022, 2022: 3481423.

[18] Turker S, Kuhnke P, Eickhoff S B, et al. Cortical, subcortical, and cerebellar contributions to language processing: A meta-analytic review of 403 neuroimaging experiments [J]. Psychological bulletin, 2023.

***[19] Yao J, Liu X, Lu X, Lu X, Xu C, Chen H, Zhang Y. Changes in white matter microstructure related to non-linguistic cognitive impairment in post-stroke aphasia [J]. Neurological research, 2020, 42(8): 640-8.***

***[20] Siqi Li, Zinan Yuan, Yuexiu Li, Yang Liu, Yumei Zhang.*** ***Abnormal resting-state neural activities of language and non-language cognitive function impairments in stroke patients with aphasia: A cross-sectional study.*** ***Clinical Neurology and Neurosurgery .2025; 251 108849.***

***[21] Liu Q, Zhang Y, Liu C, Chen Y, Zhang Y. Reduced cerebral blood flow and cognitive dysfunction following isolated cerebellar infarction: two case reports. The Journal of international medical research. 2024;52(3):3000605241235848.***

***[22] Zhang W, Fu W, Zhang Y. Association of Cerebral Hypoperfusion and Poor Collaterals with Cognitive Impairment in Patients with Severe Vertebrobasilar Artery Stenosis. Journal of Alzheimer's disease reports. 2024;8(1):999-1007.***

***[23] 苑梓楠, 李思奇, 陈心雅, 张玉梅. 卒中后运动性失语症患者的语言损伤特点分析[J]. 中国卒中杂志, 2023, 18(11): 1238-1247.***

[24] Lefaucheur J P, Aleman A, Baeken C, et al. Evidence-based guidelines on the therapeutic use of repetitive transcranial magnetic stimulation (rTMS): An update (2014-2018) [J]. Clin Neurophysiol, 2020, 131(2): 474-528.

[25] Ntasiopoulou C, Nasios G, Messinis L, et al. Repetitive Transcranial Magnetic Stimulation in Post-stroke Aphasia: Comparative Evaluation of Inhibitory and Excitatory Therapeutic Protocols: Narrative Review [J]. Advances in experimental medicine and biology, 2023, 1425: 619-28.

[26] Anglade C, Thiel A, Ansaldo AI. The complementary role of the cerebral hemispheres in recovery from aphasia after stroke: a critical review of literature [J]. Brain Inj, 2014, 28: 138-145.

[27] Datta A, Baker J M, Bikson M, et al. Individualized model predicts brain current flow during transcranial direct-current stimulation treatment in responsive stroke patient [J]. Brain Stimul, 2011, 4: 169-174.

[28] Turkeltaub P E, Swears M K, Dmello A M, et al. Cerebellar tDCS as a novel treatment for aphasia? Evidence from behavioral and resting-state functional connectivity data in healthy adults [J]. Restor Neurol Neurosci, 2016, 34(4): 491-505.

***[29] 陈仁吉. 言语康复指南 [M]. 人民卫生出版社. 2023.09. ISBN: 9787117350662.***

[30] Xing S, Lacey E H, Skipper-Kallal L M, et al. Right hemisphere grey matter structure and language outcomes in chronic left hemisphere stroke [J]. Brain, 2016, 139: 227-241.

[31] Skipper-Kallal L M, Lacey E H, Xing S, et al. Right Hemisphere Remapping of Naming Functions Depends on Lesion Size and Location in Poststroke Aphasia [J]. Neural Plast 2017, 2017: 8740353.

[32] Breining B L, Sebastian R. Neuromodulation in post-stroke aphasia treatment [J]. Curr Phys Med Rehabil Rep, 2020, 8: 44-56.

[33] Marangolo P, Fiori V, Caltagirone C, et al. Transcranial Cerebellar Direct Current Stimulation Enhances Verb Generation but Not Verb Naming in Poststroke Aphasia [J]. J Cogn Neurosci, 2018, 30(2): 188-199.

[34] Sebastian R, Kim J H, Brenowitz R, et al. Cerebellar neuromodulation improves naming in post-stroke aphasia [J]. Brain communications, 2020, 2(2): fcaa179.

[35] Demarco A T, Dvorak E, Lacey E, et al. An Exploratory Study of Cerebellar Transcranial Direct Current Stimulation in Individuals With Chronic Stroke Aphasia [J]. Cognitive and behavioral neurology: official journal of the Society for Behavioral and Cognitive Neurology, 2021, 34(2): 96-106.

[36] Georgiou AM, Kambanaros M. The Effectiveness of Transcranial Magnetic Stimulation (TMS) Paradigms as Treatment Options for Recovery of Language Deficits in Chronic Poststroke Aphasia. Behavioural neurology. 2022; 2022: 7274115.

[37] Huang G, Wang X, Li T, et al. Differential Effects of Continuous Theta Burst Stimulation over the Bilateral and Unilateral Cerebellum on Working Memory [J]. Cerebellum (London, England), 2024, 23(6): 2360-71.

***[38] Liu Q, Liu Y, Zhang Y. Effects of Cerebellar Non-Invasive Stimulation on Neurorehabilitation in Stroke Patients: An Updated Systematic Review. Biomedicines. 2024;12(6).***

[39] Casula E P, Pellicciari M C, Ponzo V, et al. Cerebellar theta burst stimulation modulates the neural activity of interconnected parietal and motor areas [J]. Sci Rep, 2016, 6: 36191.

[40] 胡雪艳, 江晓峰, 山磊, 杨凌宇，陈予东，马琳，刘丽旭，张通. 低频和高频重复经颅磁刺激对脑卒中后非流畅性失语的影响[J]. 中国康复理论与实践, 2023, 29(3): 249-255.

[41] Barwood CH, Murdoch BE, Riek S, et al. Long term language recovery subsequent to low frequency rTMS in chronic non-fluent aphasia[J]. NeuroRehabilitation, 2013, 32(4): 915-928.

[42] Zhang J, Zhong D, Xiao X, et al. Effects of repetitive transcranial magnetic stimulation (rTMS) on aphasia in stroke patients: a systematic review and meta-analysis[J]. Clin Rehabil.
